# Genome wide association study for growth in Pakistani dromedary camels using genotyping-by-sequencing

**DOI:** 10.5713/ab.22.0181

**Published:** 2022-11-14

**Authors:** Sajida Sabahat, Asif Nadeem, Rudiger Brauning, Peter C. Thomson, Mehar S. Khatkar

**Affiliations:** 1 Institute of Biochemistry and Biotechnology, University of Veterinary and Animal Sciences, Syed Abdul Qadir Jilani (Outfall) Road, Lahore 54000, Pakistan; 2 The University of Sydney, School of Life and Environmental Sciences, 425 Werombi Road, Camden NSW 2570, Australia; 3 Present address: Department of Biotechnology, Virtual University of Pakistan, Lahore 54000, Pakistan; 4 AgResearch, Invermay Agricultural Centre, 176 Puddle Alley, Mosgiel 9092, New Zealand; 5 The University of Sydney, Sydney School of Veterinary Science, University of Sydney, Camden NSW 2570, Australia

**Keywords:** Dromedary, Growth, Genome-wide Association Study (GWAS), Lassi, Marecha

## Abstract

**Objective:**

Growth performance and growth-related traits have a crucial role in livestock due to their influence on productivity. This genome-wide association study (GWAS) in Pakistani dromedary camels was conducted to identify single nucleotide polymorphisms (SNPs) associated with growth at specific camel ages, and for selected SNPs, to investigate in detail how their effects change with increasing camel age. This is the first GWAS conducted on dromedary camels in this region.

**Methods:**

Two Pakistani breeds, Marecha and Lassi, were selected for this study. A genotyping-by-sequencing method was used, and a total of 65,644 SNPs were identified. For GWAS, weight records data with several body weight traits, namely, birthweight, weaning weight, and weights of camels at 1, 2, 4, and 6 years of age were analysed by using model-based growth curve analysis. Age-specific weight data were analysed with a linear mixed model that included fixed effects of SNP genotype as well as sex.

**Results:**

Based on the *q*-value method for false discovery control, for Marecha camels, five SNPs at *q*<0.01 and 96 at *q*<0.05 were significantly associated with the weight traits considered, while three (*q*<0.01) and seven (*q*<0.05) SNP associations were identified for Lassi camels. Several candidate genes harbouring these SNP were discovered.

**Conclusion:**

These results will help to better understand the genetic architecture of growth including how these genes are expressed at different phases of their life. This will serve to lay the foundations for applied breeding programs of camels by allowing the genetic selection of superior animals.

## INTRODUCTION

The camel is an important livestock species in many regions of the world, and is used for meat, milk, skin production, as well as a means of transport, entertainment, racing competitions, dancing and beauty shows. It is adapted to extremely harsh environments [1], and is well placed to cope with climate change. While providing financial benefit, often to economically-disadvantaged farmers, the camel has a substantial cultural value within rural communities. There are three species of camel, the dromedary (*Camelus dromedarius*) from south Asia, the Middle East and North/East Africa, the Bactrian camel (*Camelus bactrianus*) from central Asia, and wild Bactrian camel (*Camelus ferus*) from China and Mongolia [2]. However, the dromedary is by far the most common of the species and is the focus of the current paper.

Unlike other livestock species, little sy stematic breed improvement has been undertaken on the camel. Further, the genetic diversity has also not been explored thoroughly like other livestock species, and more work on this area has been conducted on the Bactrian camel than the dromedary [3,4]. The camel has a substantial potential for genetic improvement, particularly for meat and milk products. Given the adaptations of camels to be productive in environments too harsh for other livestock species, and with the urgency of climate change, it is timely to allocate resources to undertake genetic improvement programs in the camel. Thus, it is vital to study the genetic diversity and conserve germplasm resources of the camel [5,6].

Pedigree-based and genomic selection have the potential to play a major role for genetic improvement in the camel, however, it is also important to identify genes and genomic regions associated with growth and production traits, for understanding the genetic architecture of the traits and improving accuracy of selection. Genome-wide association studies (GWAS) have become routine for discovering the genes and narrow genomic regions associated with traits of interest. However, to conduct a GWAS, in addition to phenotypic records, the availability of sufficiently dense genetic markers is a prerequisite. High-density single nucleotide polymorphism (SNP) arrays have become available for several species and made a substantial contribution to breeding, and mapping disease traits in domesticated animals.

Currently no SNP chip is available for the dromedary camel. However, genotyping-by-sequencing (GBS) can be used to genotype a large number of SNPs with a much lower cost as compared to developing a SNP chip. Recently we published a study on SNP discovery and genetic diversity in the dromedary camel using this GBS technique [7]. Sequencing as compared with SNP chips can provide a range of additional insights into genetic architecture, including information about existence of SNPs, copy number variation, and insertions/deletions (indels). Due to reduction in sequencing cost, it is now feasible to sequence and genotype all individuals in a population and then perform GWAS. This has particular appeal for livestock improvement in non-traditional species such as the camel.

A small number of studies have been conducted on ge netic associations with traits in camels. Almutairi et al [8] reported a quantitative genetic analysis of growth and milk production traits of a Saudi camel population based on phenotypic and pedigree data, reporting on heritability estimates and other genetic parameters. Further, several studies have investigated trait associations with specific candidate genes. For example, Afifi et al [9] have investigated the association between the growth hormone gene and body weight in dromedary camels, while Almathen et al [10] reported associations of the melanocortin 1 receptor (*MC1R*) and agouti signalling protein (*ASIP*) genes with coat colour in dromedaries. Guo et al [11] published a GWAS on haematological traits in the Bactrian camel in China. Recently, Bitaraf Sani et al [12] presented a GWAS for an Iranian dromedary population for birth weight and average growth. However, there is no study, that the authors are aware of, in the dromedary camel, that undertake a GWAS across a range of weight-for-age traits.

The current study was designed with the objectives i) to undertake a GWAS to identify SNPs associated with growth at specific camel ages, and ii) for key significant SNPs identified in the GWAS, to investigate in detail how their effects change over the life of a camel. The analyses were performed separately for two breeds of dromedary camels commonly found in Pakistan, namely Marecha and Lassi. This is the first GWAS undertaken on dromedary camels in this region, and builds upon previous research on these two breeds in relation to growth and genetic diversity [7,13].

## MATERIALS AND METHODS

### Ethical statement

The study was undertaken in compliance with the institutional guidelines of the Ethics Review Committee of the University of Veterinary and Animal Sciences, Lahore Pakistan.

### Sample collection

Blood samples and weight records of Marecha camels (n = 70) were collected from the Camel Breeding and Research Station Rakh Mahni, Bhakkar, Punjab Pakistan (31°33′39.81″ N, 71°50′33.15″ E), that were subsequently genotyped, consisting of 53 females and 17 males. In addition, 27 female and two male Lassi camels (n = 29) were sampled from private farmers in Lasbela, Baluchistan Pakistan (25°44′59.99″ N, 66°34′59.99″ E). Up to four years weight records (approximately monthly) were collected for each camel, as well as birth and weaning weights, however the age of recording of weights during the four-year data period differed between camels.

### Genotyping

Blood samples were sent to AgResearch New Zealand ( https://www.agresearch.co.nz/) for DNA extraction and genotyping using a GBS method, specifically using an Illumina HiSeq 2500 utilising V4 chemistry and 1×100 single end sequence reads. Further details of sample collection and genotyping were described in Sabahat et al [7,13].

Using the GBS approach, 65,644 SNPs were identified. Reads were mapped onto the dromedary camel chromosomal-level draft assembly CamDro3 (https://www.ncbi.nlm.nih.gov/assembly/GCF_000803125.2). Of these, 60,831 SNPs were mapped to chromosomes. Prior to GWAS, a further filtering to remove SNPs with minor allele frequency <0.01 and call rate <0.5 was made, and only autosomal SNPs were retained resulting in 27,973 SNPs for analysis. Remaining missing SNPs were then imputed using Beagle version 5.2 using the default parameters [14]. In addition, the samples with a call rate of <0.3 were excluded, resulting in 67 Marecha and all 29 Lassi camels. Genotypes from the GBS data were recoded into the number of copies of the minor allele (0, 1, or 2).

### Statistical analysis

Phenotypes for the association analyses were birth weight and weaning weight. In addition, since weights were not available at the same set of ages of each camel, growth model predictions were used as phenotypes, based on predictions at 1, 2, 4, and 6 years of age. A linear mixed approach using splines was used for the model fitting, producing animal-specific growth curves. Details are provided in Sabahat et al [13].

Genome-wide association analysis between the weight traits and SNPs was conducted by fitting a linear mixed model at each SNP position. The model contains a fixed effect for sex as well as the SNP under investigation, although Sex was dropped from the model for the 4-yr and 6-yr Marecha dataset and all Lassi datasets because of the lack of male camels of that age, no analysis of 6-yr Lassi was undertaken because of limited records. Note that a ‘dosage’ model was considered, i.e., the number of copies of the minor allele (0, 1, or 2) was fitted as a quantitative variable, i.e. an additive model was fitted at each SNP. A polygenic term for each camel was fitted as a random effect, these being linked to relationship matrix determined from the GBS count data [7,15]. The mathematical form of the model is **y** = **Xβ**+**Zu**+**ɛ** where **y** is the vector of observations of the trait, **X** is the model matrix associated with the fixed effects, **β** is the set of fixed effects, **Z** is a model matrix associated with the random effects, simply the identity matrix **I** here, **u** is the vector of polygenic random animal effects, assumed $u\sim N(0,\sigma_{A}^{2}G)$ where $\sigma_{A}^{2}$ is the additive genetic variance and **G** is the GBS-based genomic relationship matrix. The term **ɛ** is a vector of random errors, assumed mutually independent, $ɛ\sim N(0,\sigma_{ɛ}^{2}I)$. The linear mixed model was fitted using ASReml-R [16].

Significance of each SNP was assessed using a Wald F-test, and a false discovery are evaluated for each test using the *q*-value method, implemented using the ‘qvalue’ function in R [17]. Results were visualised using a Manhattan plot. The *q*-values and thresholds were evaluated by pooling all GWAS scans within a breed. These plots identified ‘suggestive’ and ‘significant’ associations at *q*<0.1, and *q*<0.05 respectively, although only ‘significant’ associations were further investigated.

To explore any evidence of age-specific gene function for growth, selected SNPs identified in the GWAS results were assessed. Separate association analyses were conducted between the model-based weights over a fine range of ages (Marecha: 0 to 6 yr, Lassi: 0 to 4 yr, in steps of 0.25 yr) and the selected SNP. The same statistical model as used in the above GWAS was used here.

### Bioinformatic analysis

The gene annotation information for the CamDro3 assembly was downloaded from https://www.ncbi.nlm.nih.gov/assembly/GCF_000803125.2. The genes harbouring the SNPs were extracted based on the start and end positions of the genes. For each SNP where a significant trait association was identified, GeneID, gene symbol, genomic_accession, start and end positions of the genes were obtained.

## RESULTS

### Descriptive statistics of body weights

Table 1 shows descriptive statistics of body weights of the different age classes for Marecha and Lassi camel breeds, together with the sample sizes. There is not a substantial difference in the weights between the two breeds, further details of the growth and growth curve modelling are shown in Sabahat et al [13]. Mean age of weaning for Marecha camels is 210 days (range, 63 to 518 days; standard deviation [SD], 113 days), while Lassi camels have a mean age at weaning of 237 days (range, 59 to 396 days; SD, 83 days). As is shown, the number of samples available decrease with increasing age, and while all Lassi camels have corresponding genotypic data, about 62% of Marecha camels have genotypic data.

### Overview of genome-wide associations

**Figure 1:**
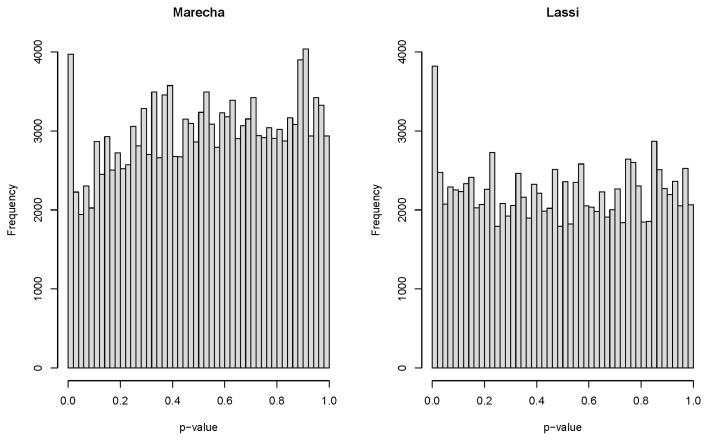
Combined distributions of p-values for genome-wide association study of weights traits at different ages, shown separately for Marecha and Lassi camel breeds.

Table 2 shows the number of associations detected across the breeds and age groups at threshold levels of *q*<0.01, *q*<0.05, and *q*<0.1. While a large number of associations were detected for some breed×age groups (e.g. four years of age for Marecha, two years of age for Lassi), associations were not found in all groups (e.g. birth weight, weaning weight, and weights at one year for Marecha). Figure 1 shows the distribution of p-values for the genome-wide association tests, separately for each breed, although all the age class traits have been combined into these plots. The excess of small p-values supports evidence for the existence of significant associations in both breeds. A complete list of associations, including SNP and linkage group specifications, age class, significance levels, and allele substitution effects (regression coefficients) is included in Supplementary Table S1. Manhattan plots are provided only when associations with *q*<0.05 were detected.

### Genome-wide associations for Marecha camels

**Figure 2:**
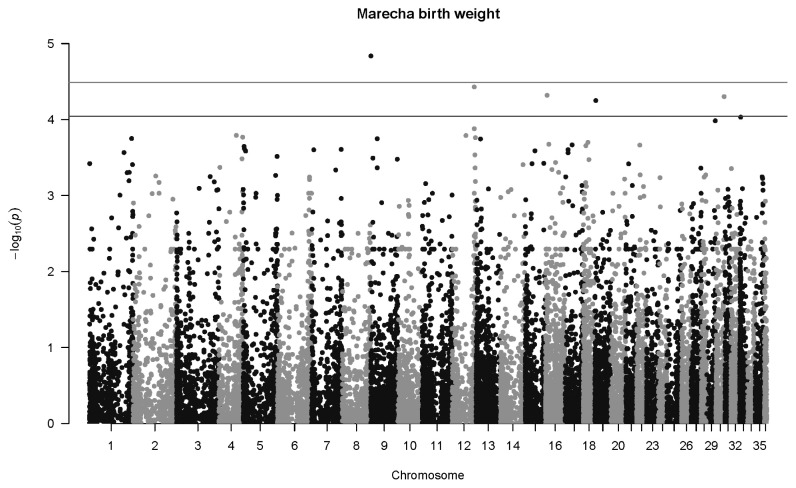
Manhattan plot for genome-wide associations with birth weight in Marecha camels. The horizontal lower line is drawn at a threshold of *q* = 0.10 and the upper line at a threshold of *q* = 0.05. The most significant association is in Chromosome 9 at 521,945 bp (SNP 27264_39, p = 1.5×10^−5^). SNP, single nucleotide polymorphisms.

**Figure 3:**
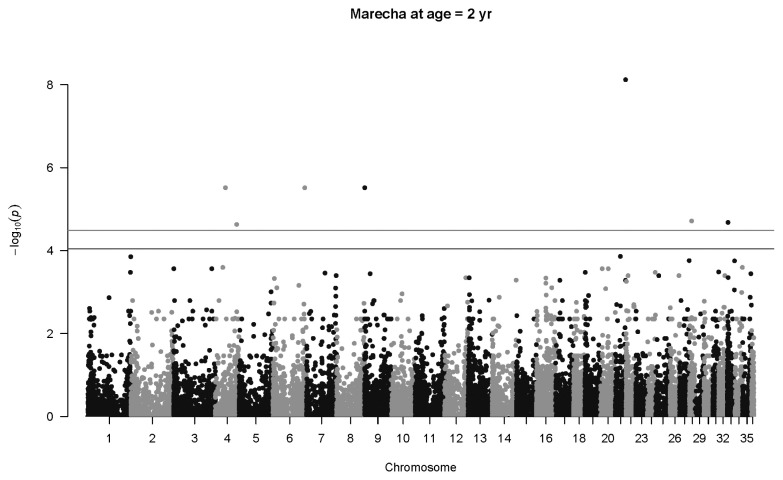
Manhattan plot for genome-wide associations with weight at two years in Marecha camels. The horizontal lower line is drawn at a threshold of *q* = 0.10 and the upper line at a threshold of *q* = 0.05. The most significant association is in Chromosome 21 at 29,903,214 bp (SNP 154340_31, p = 7.6×10^−9^).

For Marecha camels, there was one significant association for birth weight, with SNP 27264_39 located on Chromosome 9 (p = 1.5×10^−5^, *q* = 0.029), with an allelic substitution effect (*b*) of −4.43±1.02 kg (regression parameter estimate± standard error [SE]). The Manhattan plot for associations with Marecha birth weight at is shown in Figure 2. No significant associations were detected for weaning weight, nor at one year of age. However, at two years of age, the most significant association was detected for SNP 154340_31 located on Chromosome 21 (p = 7.6×10^−9^, *q* = 0.00057), with an allelic substitution effect (*b*) of 144.3±25.0 kg (regression parameter estimate±SE). The Manhattan plot for this is shown in Figure 3.

**Figure 4:**
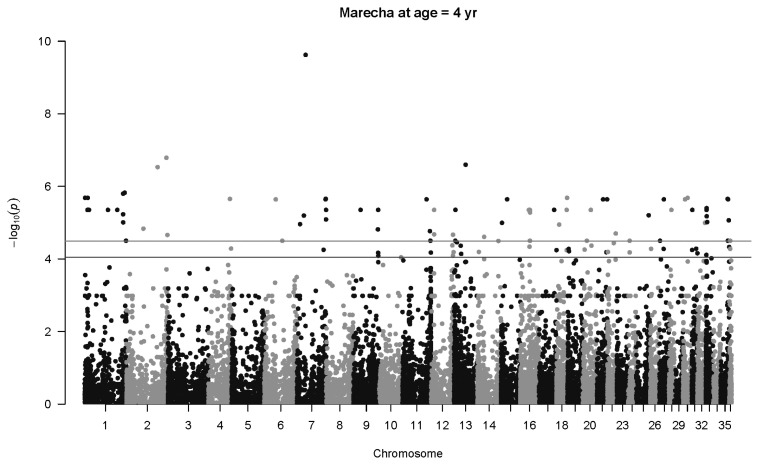
Manhattan plot for genome-wide associations with weight at four years in Marecha camels. The horizontal lower line is drawn at a threshold of q = 0.10 and the upper line at a threshold of q = 0.05. The most significant association is in Chromosome 7 at 25,542,049 bp (SNP 20732_39, p = 2.4×10^−10^). SNP, single nucleotide polymorphisms.

A large number of highly significant associations were mapped for four-year old Marecha camels four with *q*<0.01 (Figure 4). The most significant association was for SNP 20732_39 in Chromosome 7 (p = 2.4×10^−10^, *q* = 3.6×10^−5^, *b* = −159.2±25.1 kg). Two other highly significant associations were detected on Chromosome 2 and another on Chromosome 13. No significant associations were detected at six years of age, likely due to fewer observations in this age category.

### Genome-wide associations for Lassi camels

**Figure 5:**
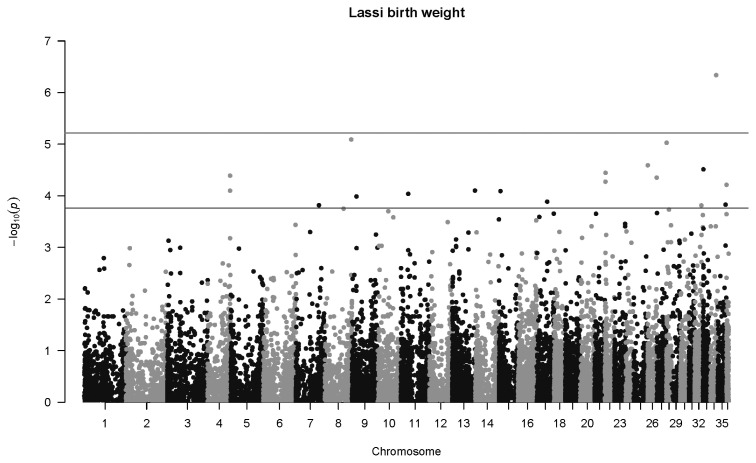
Manhattan plot for genome-wide associations with birth weight in Lassi camels. The horizontal lower line is drawn at a threshold of *q* = 0.10 and the upper line at a threshold of *q* = 0.05. The most significant association is in Chromosome 34 at 17,837,742 bp (SNP 84745_29, p = 4.6×10^−7^). SNP, single nucleotide polymorphisms.

**Figure 6:**
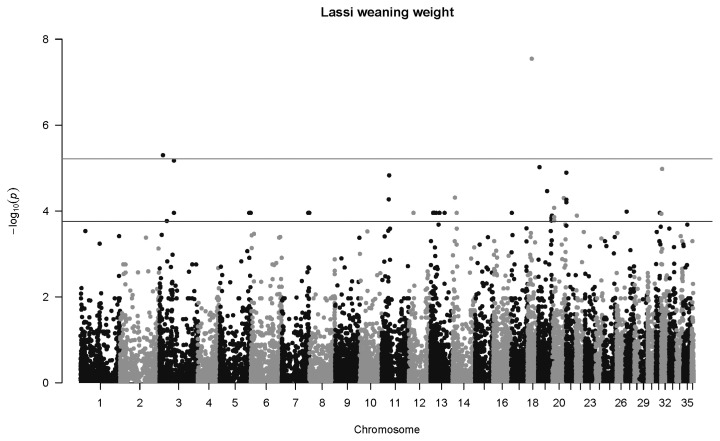
Manhattan plot for genome-wide associations with weaning weight in Lassi camels. The horizontal lower line is drawn at a threshold of *q* = 0.10 and the upper line at a threshold of *q* = 0.05. The most significant association is in Chromosome 18 at 13,190,875 bp (SNP 149372_20, p = 2.9×10^−8^). SNP, single nucleotide polymorphisms.

For birth weight in Lassi camels, one highly significant association was detected at *q*<0.01, and 25 were mapped at *q*<0.1 (Figure 5). The most significant association was SNP 84745_29 in Chromosome 34 (p = 4.6×10^−7^, *q* = 0.013, *b* = −5.06±1.00 kg). One highly significant association was mapped for weaning weight in Lassi camels (Figure 6), namely SNP 149372_20 on Chromosome 18 (p = 2.9×10^−8^, *q* = 0.0018, *b* = −20.9±3.8 kg).

**Figure 7:**
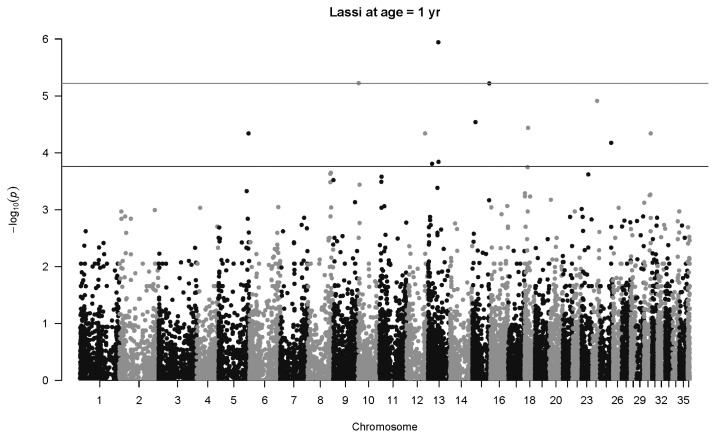
Manhattan plot for genome-wide associations with weight at one year in Lassi camels. The horizontal lower line is drawn at a threshold of *q* = 0.10 and the upper line at a threshold of *q* = 0.05. The most significant association is in Chromosome 13 at 32,716,593 bp (40507_65, p = 1.1×10^−6^).

**Figure 8:**
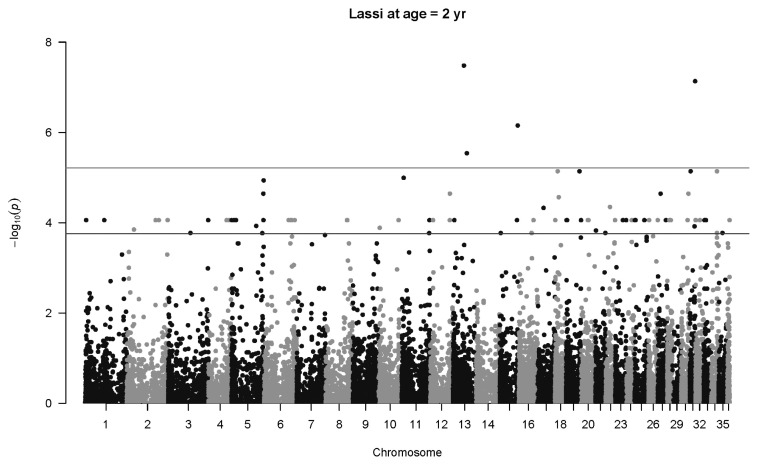
Manhattan plot for genome-wide associations with weight at two years in Lassi camels. The horizontal lower line is drawn at a threshold of *q* = 0.10 and the upper line at a threshold of *q* = 0.05. The most significant association is in Chromosome 13 at 32,716,593 bp (40507_65, p = 3.3×10^−8^).

There is one highly significant SNP associations for Lassi weight at one year (Figure 7), namely SNP 40507_65 in Chromosome 13 (p = 1.1×10^−6^, *q* = 0.021, *b* = 50.6±10.4 kg). Another SNP less than 1 Mb upstream (141357_76) also has an association with body weight at age two years (p = 0.00015, *q* = 0.093, *b* = 43.8±11.5 kg). The same SNP mentioned above, 40507_65, was also highly significant at two years of age (p = 3.3×10^−8^, *q* = 0.0018, *b* = 107.4±19.4 kg), as seen in Figure 8. Three other significant associations were detected, notably SNP 79310_43 on Chromosome 31 (p = 7.3×10^−8^, *q* = 0.0023, *b* = 69.9±13.0 kg). No significant associations were detected at four years of age for Lassi camels, although the smaller number of Lassi camels at this age should be taken into consideration.

### Age-specific associations

An analysis was conducted to investigate the effect of specific SNPs across the age span of camels. While most of the SNP associations were at a specific age class (308, at *q*<0.10), there were ten SNPs with association with two age classes, as summarised in Table 3. Eight of these involved Lassi camels at one and two years of age. Three of these are for Marecha camels at ages two and four years, the remaining associations with Lassi camels for birth weight and Marecha camels at four years of age.

**Figure 9:**
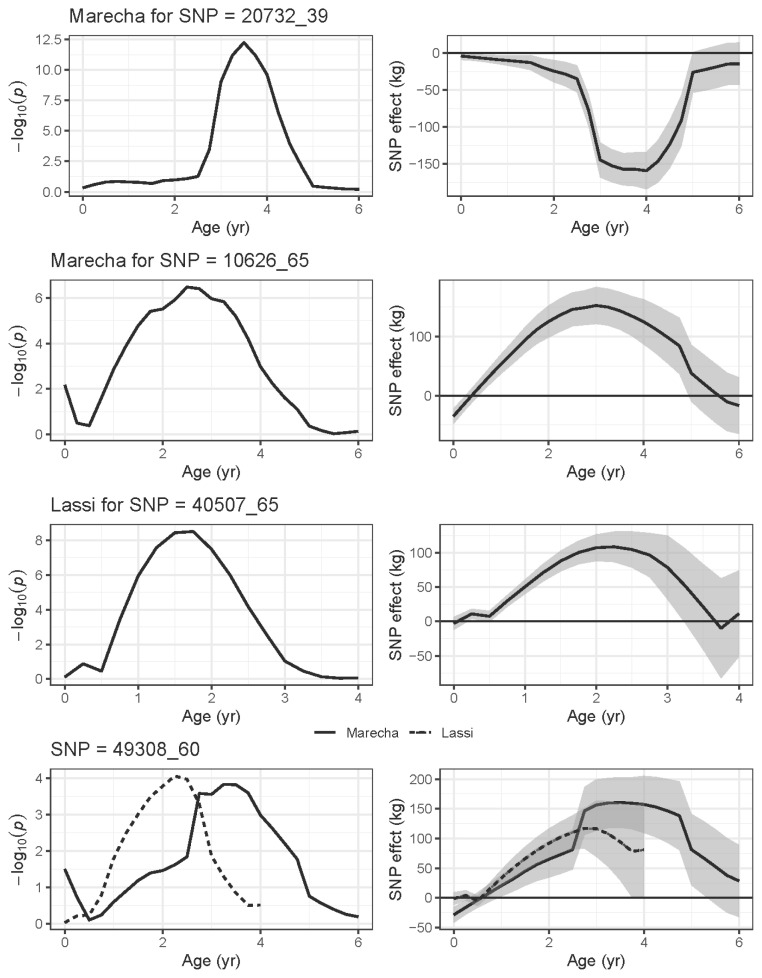
Association plots of selected SNPs from GWAS studies showing the effect of the SNP over the six (Marecha) and four (Lassi) years of the camel’s recorded growth period. The left-hand side panels show significance levels (−log_10_*p*) for the mixed model association tests at each SNP, whereas the right-hand side panels show corresponding estimated effects of the SNP displayed as regression coefficients with standard error bands (*b*±SE). The plots for SNP 493-8_60 include both breeds, as both had significant associations with weight traits (see Table 3). SNP, single nucleotide polymorphisms; GWAS, genome-wide association study.

Figure 9 show the phenotypic effect of four selected SNPs with increasing age, up to six years in Marecha and four years in Lassi camels. The panels on the left hand show the significance levels (−log_10_*p*) while the right-hand panels show the estimated effects expressed as regression coefficient with SE bands. SNP 2072_39 on Chromosome 7 was the most significant association for Marecha noted, and has strong effects from around three to five years of age. SNP 10626_65 on Chromosome 21, though only initially detected to have an association at two years, has strong effects on body weight from about one to five years of age. For Lassi camels, SNP 40507_65 on Chromosome 13 had significant associations at ages 1 yr and 2 yr (Table 3), and this is reflected in Figure 9. Finally, SNP 49308_60 on Chromosome 16 shows the significant associations for Marecha, and while it did not pass the threshold for Lassi camels, it does show some support for an earlier effect in Lassi compared with Marecha. Overall though, the plots suggests SNPs were associated with specific periods of growth rather than across the entire growth profile.

### Bioinformatic analysis and identification of associated genes

The SNPs involved in associations with the weight traits were further evaluated to identify the genes. Information including GeneID, gene symbol, genomic accession, start and end positions of the genes harbouring the significant SNPs are given in Supplementary Table S1. Out of the significant SNPs, 178 SNPs could be assigned within the known annotated genes.

## DISCUSSION

The camel is an excellent source of meat production in harsh and drought conditions. Genetic improvement of growth traits of the camel is critical to achieve increased meat and production efficiency. Therefore, it is necessary to find major genes that are associated with growth for future breeding selection. In this study, we perform GWAS of growth traits at different ages of their life, and significant association of SNPs have been identified.

GWAS is an efficient method for searching candidate genes associated with growth. However, the accuracy of GWAS relies on the population structure, as well as the existence of genome-wide linkage disequilibrium [18]. Domestic animals have a simpler genetic diversity and population structure as compared with human populations [19]. In this study, all Marecha camel samples were collected from the same farm, but a principal coordinate analysis on the SNP data revealed greater genetic diversity in that breed, compared with that Lassi camels collected from multiple farms [7]. While reduced genetic diversity may impact the ability to detect genetic associations, in this study more genetic associations were detected for Lassi compared to the genetically more diverse Marecha breed. This may be due to the genes that did show diversity in Lassi have associations with growth traits. However, this would need to be confirmed with a larger number of animals to fully evaluate the genetic architecture. The main purpose of this work was to find SNPs associated with several body weights traits, namely, birthweight, weaning weight, and weights at 1, 2, 4, and 6 years old. Growth is a complex trait, involving aspects of cell proliferation, differentiation, muscle, bone and tissue development, fat, metabolism and nutrient absorption [20]. Likely, each physiological aspect is under its own specific genetic control, so a search for associations will always be a somewhat ‘blunt tool’.

At the most stringent level of testing, ( *q*<0.01), there were five SNP associations detected for Marecha (one at 2 yr, four at 4 yr) and three associations detected for Lassi camels (one for weaning, two at 2 yr), as shown in Table 2. However, many more were detected at less stringent levels (*q*<0.05), particularly for Marecha camels at 4 yr, although the limited number of Marecha in this age group may influence this result.

In terms of effect sizes, for birthweight, effects sizes (allelic substitution effects) for significant SNP associations ranged up to −4.43±1.02 kg (Marecha) and −5.06±1.00 kg (Lassi), i.e. up to ~10% of average birthweight. For weaning weight, effect sizes ranged up to 20.9±3.8 kg (Lassi), i.e. ~20% of mean weaning weight (No significant associations were detected for weaning weight in Marecha camels). Maximum effect size of significant SNPs associated with weight at specific ages tends to increase with increasing age: for Marecha: 144.3±25.0 kg at 2 yr, and −159.2±25.1 kg at 4 yr, and for Lassi: 50.6±10.4 kg at 1 yr, and 107.4±19.4 kg at 2 yr. One explanation for the relatively large number of associations at the older groups may be that the effect sizes in these older cohorts are relatively larger, increasing their power of detection. However, the small sample sizes means that these results need to be treated with caution.

Also, of note was the existence of age-specific associations. The overwhelming majority of associations were found to be age-specific. Similarly, a study by Hadjipavlou and Bishop [21] in Scottish Blackface sheep reported quantitative trait loci to be associated over specific age ranges. Nonetheless, some SNP were associated with weights at more than one age in the current study, particularly for Lassi camels with several SNP associations at one and two years of age (Table 3, Figure 9). These SNPs that show strong association with growth can be used as candidate gene in breeding program, perhaps incorporating a panel of genes that might respond at different ages. Nevertheless, it should again be considered that the associations at older ages are less clear, due to the reduced sample sizes. The significant SNPs were within 142, out of which 128 were protein coding and several of them have interesting biological functions relevant to the growth and metabolism. Further confirmation of these association with a larger sample size may be warranted before delving into functions of these candidate genes.

The current work builds on the study by Bitaraf Sani et al [12] who reported the results of a GWAS on 96 dromedaries of several breeds in Iran. When the results of the Bitaraf Sani et al [12] paper and the current study are compared, it was noted that a number of SNP associations within a ±1 Mb window were in common, notably on chromosomes 7, 11, 18, 25, 31, and 33 (Supplementary Table S2). For example, there were four neighbouring SNPs on chromosome 18 (~ 30.3 Mb) relating to Marecha body weight in the current study which were within 0.6 Mb of eight neighbouring SNPs in the Iranian study of SNPs associated with body weight for which the authors suggested candidate genes of dexamethasone-induced protein (*DEXI*), testis-specific Y-encoded-like protein 4 (*TSPYL4*) and Class II transactivator (*CIITA*). The current study provides a GWAS for body weight at a range of specific ages, and also tracks the effects of selected SNPs across ages of the camel. The novelty of this work has been enhanced by using the output from the growth curve analysis as additional weight phenotypes to detect effects of SNPs at different ages of animals Nevertheless there are some limitations to the current study, one major issue being the relatively small number of animals in the study. While adequate for the genetic diversity and population structure reported in Sabahat et al [7], this is not ideal for a GWAS. Given the genetic difference between the two breeds considered here, it was necessary to perform separate GWAS as Marecha and Lassi, and this would reduce the power of detecting associations compared with a combined analysis. Nevertheless, the work presented here can be considered as laying the foundations for a more comprehensive map. This will facilitate development of marker-assisted selection approaches, and with increase in camel numbers genotyped, the possibility of a genomic selection approach for breed improvement in camels could be considered, as is currently conducted in other livestock species.

## CONCLUSION

In summary, our GWAS identified SNPs associated with body weight of *Camelus dromedarius*. Based on growth models, it was possible to evaluate genetic variation in weight across different ages, and it is apparent that genes are expressed at different ages during the growth of camels. Several genes harbouring the significant SNPs linked with growth traits were detected and some of them have interesting biological functions. However, future studies are required to confirm and target these areas that could provide further knowledge about genetic architecture of growth traits. These findings should facilitate and contribute to marker-assisted selection breeding program of the camel.

## Figures and Tables

**Table 1:** Descriptive statistics on body weights of Marecha and Lassi camels^1)^

Age group	Marecha			Lassi		
	
	Mean (kg)	SD (kg)	n^2)^	Mean (kg)	SD (kg)	n^2)^
Birth	40.5	5.4	108 (67)	40.8	5.2	29 (29)
Weaning	105.0	15.2	108 (67)	102.0	19.1	29 (29)
1 yr	139.8	28.9	107 (66)	133.8	22.6	29 (29)
2 yr	228.2	50.7	89 (56)	231.6	45.1	28 (28)
4 yr	367.4	87.4	59 (35)	356.5	77.2	11 (11)
6 yr	446.2	96.4	48 (29)	448.4	27.1	4 (4)

SD, standard deviation; GWAS, genome-wide association study.

1) Actual birth weights and weaning weights are used, and model-based weight predictions are used for the 1, 2, 4 and 6-yr classes

2) Total number of phenotypic records, with the number of records with corresponding genotypes used in the GWAS shown in parentheses.

**Table 2:** Significant associations of single nucleotide polymorphisms with weight traits from mixed model tests1)

Age group	Marecha			Lassi		
	
	*q*<0.01	*q*<0.05	*q*<0.1	*q*<0.01	*q*<0.05	*q*<0.1
Birth	0	1	5	0	1	25
Weaning	0	0	0	1	1	41
1 yr	0	0	0	0	1	13
2 yr	1	7	7	2	4	115
4 yr	4	89	122	0	0	0
6 yr	0	0	0	-	-	-

1) The number of associations is shown for varying false-discovery rate (*q*-value) thresholds.

**Table 3:** SNPs with significant associations (*q*<0.10) with two age class weight traits from mixed model tests

SNP	Chrom.	Pos. (bp)	p-value	*q*	*b*±SE^1)^ (kg)	Breed	Age
176392_39	5	95,107,644	4.6×10^−5^	0.071	54.9±13.5	Lassi	1 yr
			2.3×10^−5^	0.071	111.6±26.3	Lassi	2 yr
29880_61	10	3,579,814	6.0×10^−6^	0.051	48.6±10.7	Lassi	1 yr
			1.3×10^−4^	0.086	87.5±22.9	Lassi	2 yr
36646_9	12	58,753,060	4.6×10^−5^	0.071	54.9±13.5	Lassi	1 yr
			2.3×10^−5^	0.071	111.6±26.3	Lassi	2 yr
42394_15	13	8,748,123	3.5×10^−5^	0.053	−129.3±31.2	Marecha	4 yr
			1.1×10^−4^	0.076	−40.2±10.4	Lassi	Weaning
40507_65	13	32,716,593	1.1×10^−6^	0.021	50.6±10.4	Lassi	1 yr
			3.3×10^−8^	0.0018	107.4±19.4	Lassi	2 yr
46819_74	15	53,476,080	6.0×10^−6^	0.051	25.0±5.5	Lassi	1 yr
			7.0×10^−7^	0.016	52.1±10.5	Lassi	2 yr
108957_35	18	10,980,151	3.7×10^−5^	0.071	−16.6±4.0	Lassi	1 yr
			2.7×10^−5^	0.071	−33.7±8.0	Lassi	2 yr
62615_16	20	5,746,481	5.5×10^−5^	0.072	150.6±37.4	Marecha	4 yr
			1.7×10^−4^	0.098	−17.7±4.7	Lassi	Weaning
78060_23	30	21,350,149	4.6×10^−5^	0.071	54.9±13.5	Lassi	1 yr
			2.3×10^−5^	0.071	111.6±26.3	Lassi	2 yr
78060_33	30	21,350,159	4.6×10^−5^	0.071	54.9±13.5	Lassi	1 yr
			2.3×10^−5^	0.071	111.6±26.3	Lassi	2 yr

SNP, single nucleotide polymorphisms.

1) *b*±SE is the estimated allelic substitution effect (regression coefficient, kg) along with its standard error.

## SUPPLEMENTARY MATERIAL

Supplementary Table S1. The CSV file ‘Supporting Table 1’ contains information of each SNP association with any of the age-class weight traits. A total of 331 associations are listed in the file based on a *q*-value (adjusted p-value) of *q*<0.1. Column entries of the file are: SNP: SNP name; chr: chromosome number; pos: SNP position in chromosome (bp); p: p-value for SNP association test; b: allele substitution effect (i.e. regression coefficient); se.b: standard error of effect; q: FDR-adjusted p-value; Breed: camel breed (Marecha or Lassi); Age: age (BirthWt, WeaningWt, 1, 2, and 4 yr); flank (basepair sequence flanking to the SNP); feature (if SNP is within a gene) ; class (type of sequence); assembly; genomic_accession; start (start position of genomic_accession); end (start position of genomic_accession); symbol (sequence symbol); GeneID.

Supplementary Table S2. The PDF file ‘Supporting Table 2’ contains a list of SNP associations mapped in the current study with SNPs that were within 1 Mb of those mapped in the study on Iranian breeds of dromedary by Bitaraf Sani et al [12].

Raw genotypic data The raw genotypic data on 103 camel samples genotyped for 65,644 SNPs mapped onto the dromedary camel chromosomal-level draft assembly CamDro3 (https://www.ncbi.nlm.nih.gov/assembly/GCF_000803125.2) are provided in supporting file “CamDro3.vcf” in VCF format. The details on these samples are provided in the raw phenotypic data file.

Raw phenotypic data The raw phenotypic data on 137 camels are provided in the CSV file. “final data sheet gbs-adj-v2.csv”); note that not every phenotyped camel was genotyped. The field ‘sample_gbs’ is the camel ID, allowing linkage to the genotypic record. In addition to key information on each camel (date of birth, breed and sex), birth weight, weaning weight, and monthly body weights (kg) recorded between July 2013 and June 2017 are shown.
