# Supplementary material for: Genome wide association study for growth in Pakistani dromedary camels using genotyping-by-sequencing
Source: Anim Biosci. 2022 Nov 14;36(7):1010–21. doi: 10.5713/ab.22.0181 (PMC10330985; doi:10.5713/ab.22.0181)
Supplement: Supplementary Table S1. — The CSV file ‘Supporting Table 1’ contains information of each SNP association with any of the age-class weight traits. A total of 331 associations are listed in the file based on a q-value (adjusted p-value) of q<0.1. Column entries of the file are: SNP: SNP name; chr: chromosome number; pos: SNP position in chromosome (bp); p: p-value for SNP association test; b: allele substitution effect (i.e. regression coefficient); se.b: standard error of effect; q: FDR-adjusted p-value; Breed: camel breed (Marecha or Lassi); Age: age (BirthWt, WeaningWt, 1, 2, and 4 yr); flank (basepair sequence flanking to the SNP); feature (if SNP is within a gene) ; class (type of sequence); assembly; genomic_accession; start (start position of genomic_accession); end (start position of genomic_accession); symbol (sequence symbol); GeneID. [file ab-22-0181-Supplementary-Table-1.pdf]

Table 1.

SNP	chr	pos	p	b	se.b	q
20732_39	7	25542049	2.37E-10	-159.192086	25.12904615	3.56E-05
154340_31	21	29903214	7.57E-09	144.3428303	24.98231134	0.000567393
149372_20	18	13190875	2.86E-08	-20.94149909	3.773302213	0.00184824
40507_65	13	32716593	3.31E-08	107.359614	19.43417186	0.00184824
79310_43	31	16003963	7.33E-08	69.92402445	12.99011402	0.002730151
4322_44	2	118922449	1.63E-07	78.2746368	14.94473378	0.008130308
40656_22	13	34856641	2.52E-07	153.8493769	29.83875668	0.008829766
6625_78	2	92590903	2.94E-07	143.8474075	28.05701205	0.008829766
84745_29	34	17837742	4.60E-07	-5.062176166	1.003903689	0.012834527
46819_74	15	53476080	7.03E-07	52.13047132	10.50931006	0.015718467
40507_65	13	32716593	1.14E-06	50.58197063	10.39630753	0.021271877
570_78	1	119230476	1.49E-06	-143.3035926	29.77891309	0.011508422
334_57	1	114892489	1.59E-06	97.88463226	20.39394832	0.011508422
115321_34	30	13314740	2.07E-06	-162.3276619	34.20147174	0.011508422
1843_55	1	1701525	2.07E-06	-162.3276619	34.20147174	0.011508422
3627_54	1	9347501	2.07E-06	-162.3276619	34.20147174	0.011508422
56400_89	18	31389021	2.07E-06	-162.3276619	34.20147174	0.011508422
131696_75	7	85373455	2.19E-06	-139.9425368	29.55670368	0.011508422
12191_60	4	63524351	2.22E-06	-159.7876942	33.76569425	0.011508422
126001_51	4	63531559	2.22E-06	-159.7876942	33.76569425	0.011508422
86306_46	35	20820322	2.22E-06	-159.7876942	33.76569425	0.011508422
137595_71	11	71663573	2.28E-06	205.3980089	43.4513425	0.011508422
17625_84	6	33991165	2.28E-06	205.3980089	43.4513425	0.011508422
22717_89	7	84839308	2.28E-06	205.3980089	43.4513425	0.011508422
45352_47	15	18197537	2.28E-06	205.3980089	43.4513425	0.011508422
63097_55	21	18236667	2.28E-06	205.3980089	43.4513425	0.011508422
64151_44	21	30172925	2.28E-06	205.3980089	43.4513425	0.011508422
64151_70	21	30172951	2.28E-06	205.3980089	43.4513425	0.011508422
73552_77	27	12822273	2.28E-06	205.3980089	43.4513425	0.011508422
78573_32	30	5644619	2.28E-06	205.3980089	43.4513425	0.011508422
86463_39	35	22155568	2.28E-06	205.3980089	43.4513425	0.011508422
41132_40	13	41662494	2.90E-06	62.65679906	13.39489956	0.046312112
10626_65	4	28723315	3.05E-06	125.7796528	26.95027269	0.011508422
19638_45	6	92320807	3.05E-06	125.7796528	26.95027269	0.011508422
28635_67	9	733204	3.05E-06	125.7796528	26.95027269	0.011508422
116944_17	33	2639260	3.99E-06	155.6807375	33.75557643	0.011508422
116944_75	33	2639318	3.99E-06	155.6807375	33.75557643	0.011508422
83600_42	33	2528049	3.99E-06	155.6807375	33.75557643	0.011508422
83600_63	33	2528070	3.99E-06	155.6807375	33.75557643	0.011508422
105118_52	13	4517153	4.45E-06	-173.2121808	37.74430644	0.011508422
114946_62	28	7062715	4.45E-06	-173.2121808	37.74430644	0.011508422
116925_76	33	2230791	4.45E-06	-173.2121808	37.74430644	0.011508422
121313_13	1	8793573	4.45E-06	-173.2121808	37.74430644	0.011508422
121313_65	1	8793625	4.45E-06	-173.2121808	37.74430644	0.011508422
134904_73	9	73650806	4.45E-06	-173.2121808	37.74430644	0.011508422
140164_79	12	8959317	4.45E-06	-173.2121808	37.74430644	0.011508422
140164_79	12	8959317	4.45E-06	-173.2121808	37.74430644	0.011508422
1630_10	1	13236654	4.45E-06	-173.2121808	37.74430644	0.011508422
182306_39	1	97279029	4.45E-06	-173.2121808	37.74430644	0.011508422
22983_72	7	86225622	4.45E-06	-173.2121808	37.74430644	0.011508422
25767_87	9	21258686	4.45E-06	-173.2121808	37.74430644	0.011508422
3265_50	1	69605737	4.45E-06	-173.2121808	37.74430644	0.011508422
3265_78	1	69605765	4.45E-06	-173.2121808	37.74430644	0.011508422
41287_42	13	4371517	4.45E-06	-173.2121808	37.74430644	0.011508422
41287_70	13	4371489	4.45E-06	-173.2121808	37.74430644	0.011508422
48404_35	16	25581394	4.45E-06	-173.2121808	37.74430644	0.011508422
48582_13	16	28466190	4.45E-06	-173.2121808	37.74430644	0.011508422
53418_79	17	44978708	4.45E-06	-173.2121808	37.74430644	0.011508422
56307_46	18	30302544	4.45E-06	-173.2121808	37.74430644	0.011508422
56308_47	18	30305338	4.45E-06	-173.2121808	37.74430644	0.011508422
56310_55	18	30307339	4.45E-06	-173.2121808	37.74430644	0.011508422
61358_8	20	24237122	4.45E-06	-173.2121808	37.74430644	0.011508422
79527_24	31	1942754	4.45E-06	-173.2121808	37.74430644	0.011508422
79527_27	31	1942757	4.45E-06	-173.2121808	37.74430644	0.011508422
79527_32	31	1942762	4.45E-06	-173.2121808	37.74430644	0.011508422
93592_15	1	11018325	4.45E-06	-173.2121808	37.74430644	0.011508422
7343_8	3	11093604	5.04E-06	-19.91532258	4.364476386	0.050703199

48631_19	16	29025512	5.25E-06	89.39485934	19.62800623	0.013347548
308_54	1	114593410	5.86E-06	98.80327588	21.80425678	0.014644257
29880_61	10	3579814	5.98E-06	48.64752405	10.74551446	0.050703199
46819_74	15	53476080	6.04E-06	24.95321152	5.514558491	0.050703199
71043_39	25	36921480	6.25E-06	105.7094071	23.39896477	0.01536658
20630_84	7	20351828	6.38E-06	-141.8980308	31.43996379	0.015435551
116956_24	33	2806994	6.67E-06	154.6560056	34.33727342	0.01562124
116956_71	33	2806947	6.67E-06	154.6560056	34.33727342	0.01562124
124135_31	3	45368068	6.71E-06	-33.66666667	7.477000272	0.050703199
117254_76	34	17616256	7.26E-06	115.2624269	25.69447062	0.050703199
163005_83	31	2563914	7.26E-06	115.2624269	25.69447062	0.050703199
57070_58	18	7898744	7.26E-06	115.2624269	25.69447062	0.050703199
59369_47	19	40299702	7.26E-06	115.2624269	25.69447062	0.050703199
59369_7	19	40299662	7.26E-06	115.2624269	25.69447062	0.050703199
24885_56	8	79067134	8.12E-06	-7.517913833	1.684924376	0.05339582
100018_11	7	86161349	8.15E-06	-153.8808967	34.49377266	0.018522465
22989_28	7	86238280	8.15E-06	-153.8808967	34.49377266	0.018522465
86630_62	35	24304507	8.60E-06	-91.22227964	20.5013176	0.019254373
76296_11	28	762517	9.38E-06	4.585888889	1.034972469	0.055894122
60146_74	19	5042469	9.53E-06	-22.98333333	5.190916005	0.055894122
83833_55	33	4172256	9.64E-06	117.4641599	26.54496877	0.021255464
334_15	1	114892531	9.81E-06	101.1788907	22.88395605	0.021310474
45837_63	15	2880870	1.01E-05	122.6106988	27.77309666	0.021381039
81238_14	32	22635642	1.01E-05	-151.3270991	34.27957306	0.021381039
33074_52	11	6011941	1.02E-05	50.0517798	11.33950647	0.055894122
81816_65	32	3029557	1.05E-05	-17.69536424	4.015752755	0.055894122
23066_14	7	8912545	1.10E-05	89.57266162	20.3727818	0.022890216
57105_54	18	8150234	1.13E-05	172.9841001	39.39696493	0.023196511
16334_86	5	96005258	1.15E-05	68.12144638	15.53154463	0.058630211
69340_39	24	16246529	1.23E-05	42.35368644	9.68527471	0.059530373
62907_39	21	1383804	1.28E-05	-17.95517241	4.115296516	0.0597127
27264_39	9	521945	1.46E-05	-4.42841714	1.021489329	0.029275374
5950_90	2	50424440	1.46E-05	-119.443912	27.55985676	0.029275374
102481_55	11	23772849	1.48E-05	21.86082474	5.046880583	0.066168358
28604_72	9	72708004	1.53E-05	-109.9554939	25.42675448	0.030174544
103229_45	11	81674156	1.71E-05	-128.2401878	29.82898086	0.033381957
76122_73	28	6311225	1.93E-05	64.71665166	15.14649141	0.037120783
112118_16	22	24884156	1.99E-05	-75.65981099	17.73496471	0.037747728
83492_73	33	2124114	2.09E-05	82.62895576	19.42036476	0.038890029
104266_66	12	9123376	2.10E-05	-132.2537887	31.09010573	0.038890029
37431_56	12	65066709	2.15E-05	71.84498237	16.90902765	0.039278643
4831_59	2	121671751	2.18E-05	-72.77470544	17.14132067	0.039388216
176392_39	5	95107644	2.27E-05	111.5719836	26.33800498	0.070595797
36646_9	12	58753060	2.27E-05	111.5719836	26.33800498	0.070595797
75326_42	27	8462139	2.27E-05	111.5719836	26.33800498	0.070595797
78060_23	30	21350149	2.27E-05	111.5719836	26.33800498	0.070595797
78060_33	30	21350159	2.27E-05	111.5719836	26.33800498	0.070595797
11913_91	4	61257553	2.34E-05	101.8741929	24.0838671	0.041717971
42954_54	14	19944654	2.46E-05	-96.8260471	22.95428224	0.04343757
73276_85	26	566988	2.56E-05	-7.057308649	1.676610696	0.070595797
108957_35	18	10980151	2.73E-05	-33.67386809	8.026682978	0.070595797
106806_35	15	9825662	2.90E-05	-18.42283258	4.406092281	0.070595797
83033_34	33	1415092	3.07E-05	4.596815989	1.102774322	0.070595797
114229_62	27	1559192	3.15E-05	160.046531	38.45194893	0.049219104
1270_40	1	123322908	3.15E-05	160.046531	38.45194893	0.049219104
138211_64	11	83029323	3.15E-05	160.046531	38.45194893	0.049219104
152520_52	20	12508145	3.15E-05	160.046531	38.45194893	0.049219104
167333_39	36	2347462	3.15E-05	160.046531	38.45194893	0.049219104
17972_12	6	53378628	3.15E-05	160.046531	38.45194893	0.049219104
41027_22	13	4067396	3.15E-05	160.046531	38.45194893	0.049219104
48583_12	16	28477745	3.15E-05	160.046531	38.45194893	0.049219104
48637_73	16	29050713	3.15E-05	160.046531	38.45194893	0.049219104
70035_68	24	5119713	3.15E-05	160.046531	38.45194893	0.049219104
86390_73	35	21429102	3.15E-05	160.046531	38.45194893	0.049219104
105845_60	14	62512786	3.20E-05	78.61043054	18.90265213	0.049474624
58882_11	19	28608801	3.44E-05	-20.66019417	4.98799368	0.070595797
42394_15	13	8748123	3.47E-05	-129.3413073	31.24173859	0.053133659
65578_31	22	2051520	3.59E-05	-4.741496485	1.147482053	0.070595797
108957_35	18	10980151	3.66E-05	-16.56159526	4.011756104	0.070595797

111976_67	22	18966680	3.66E-05	-146.3615095	35.45594036	0.055425874
37056_23	12	62950801	3.72E-05	3.75349837	0.91014345	0.055811829
126320_34	4	66640514	4.08E-05	-10.01027403	2.439820286	0.070595797
126320_51	4	66640531	4.08E-05	-10.01027403	2.439820286	0.070595797
12878_19	4	66635456	4.08E-05	-10.01027403	2.439820286	0.070595797
12880_85	4	66639163	4.08E-05	-10.01027403	2.439820286	0.070595797
139550_67	12	63200162	4.22E-05	89.27020057	21.79944649	0.062654574
61413_18	20	26308326	4.30E-05	-72.1336049	17.63376036	0.06314768
140774_26	13	20205394	4.34E-05	-120.9381835	29.5786126	0.06314768
72905_62	26	27087462	4.45E-05	4.541320114	1.112358329	0.070595797
65128_67	22	12892371	4.49E-05	65.31569212	16.00639112	0.070595797
65133_61	22	12977545	4.49E-05	65.31569212	16.00639112	0.070595797
176392_39	5	95107644	4.57E-05	54.90350472	13.46867032	0.070595797
36646_9	12	58753060	4.57E-05	54.90350472	13.46867032	0.070595797
78060_23	30	21350149	4.57E-05	54.90350472	13.46867032	0.070595797
78060_33	30	21350159	4.57E-05	54.90350472	13.46867032	0.070595797
107148_82	16	27421215	4.59E-05	149.123614	36.58933509	0.066168091
86699_7	35	25597425	4.69E-05	-66.22125004	16.26870785	0.066999662
52416_45	17	17680135	4.71E-05	57.89680583	14.22591695	0.070595797
107530_58	16	4741360	4.79E-05	-2.641649664	0.649757264	0.067769801
143641_5	14	7354948	4.88E-05	-18.62424242	4.586034246	0.070595797
78207_90	30	23344402	4.99E-05	2.819201917	0.695039563	0.069900927
153078_24	20	35915399	5.00E-05	-15.74963821	3.883615808	0.070595797
97221_59	4	66852778	5.18E-05	147.981208	36.56414168	0.070412434
79106_54	31	13503778	5.23E-05	67.85133068	16.77323967	0.070412434
72578_40	26	2368858	5.27E-05	-154.4700488	38.2055759	0.070412434
87284_40	36	2585944	5.27E-05	-154.4700488	38.2055759	0.070412434
73488_12	27	12158793	5.30E-05	62.47649482	15.45630791	0.070412434
59138_40	19	3612052	5.31E-05	61.32300647	15.17258611	0.070412434
155011_13	22	1858745	5.33E-05	-4.646153846	1.149817604	0.070595797
136538_40	11	22328228	5.36E-05	-22.80487805	5.64542635	0.070595797
62907_6	21	1383837	5.45E-05	-17.19791667	4.261833368	0.070595797
62615_16	20	5746481	5.53E-05	150.6234412	37.35826746	0.071978195
62615_16	20	5746481	5.53E-05	150.6234412	37.35826746	0.071978195
22133_51	7	78837909	5.60E-05	136.3453126	33.84160172	0.071978195
58849_30	19	2803506	5.61E-05	4.042708483	1.003542155	0.071978195
79115_67	31	1360486	5.68E-05	152.341638	37.84144362	0.071978195
56328_38	18	30438158	5.70E-05	57.49027469	14.28357629	0.071978195
54508_16	17	51927395	5.71E-05	89.58244683	22.25994467	0.071978195
60083_66	19	4642292	6.12E-05	103.3520315	25.78508275	0.076444721
87087_39	36	135610	6.12E-05	-3.981327841	0.993348299	0.070595797
62907_24	21	1383819	6.27E-05	-17.05172414	4.260526426	0.070595797
65404_73	22	1819895	6.39E-05	80.93021673	20.2436622	0.078009244
43407_67	14	4260563	6.39E-05	139.2029128	34.82038255	0.078009244
37819_82	12	66765410	6.50E-05	-71.67287881	17.94522362	0.078009244
154168_86	21	28027262	6.52E-05	-132.8470911	33.26767541	0.078009244
70035_31	24	5119750	6.62E-05	-125.1385045	31.36689447	0.078009244
70035_77	24	5119704	6.62E-05	-125.1385045	31.36689447	0.078009244
70035_89	24	5119692	6.62E-05	-125.1385045	31.36689447	0.078009244
34957_32	11	84415056	6.66E-05	76.47183647	19.17483998	0.078009244
158057_34	25	35832505	6.69E-05	30.3070477	7.60114105	0.070595797
28653_11	9	73481517	6.77E-05	-104.7296412	26.28703938	0.078738129
115825_14	31	17061932	6.97E-05	55.09731094	13.85248716	0.080348674
39634_75	13	21545512	7.25E-05	-132.5408319	33.40336525	0.082995489
83283_30	33	1732754	7.73E-05	61.8204814	15.64061055	0.087826677
105349_48	13	68292742	7.92E-05	-5.369230636	1.360394706	0.070595797
126317_33	4	66609052	7.97E-05	-6.515118677	1.651369675	0.070595797
106558_6	15	4700803	8.12E-05	-4.746987952	1.204560908	0.070595797
37342_81	12	64699538	8.13E-05	64.00122173	16.24165288	0.091644657
101598_75	9	74028118	8.21E-05	141.8585571	36.02216183	0.091901857
111171_17	20	5587742	8.48E-05	-20.00485437	5.089701253	0.070595797
104245_61	12	8496364	8.78E-05	147.6048595	37.63546448	0.070595797
105067_41	13	4263899	8.78E-05	147.6048595	37.63546448	0.070595797
105067_63	13	4263921	8.78E-05	147.6048595	37.63546448	0.070595797
106614_58	15	51116362	8.78E-05	147.6048595	37.63546448	0.070595797
113358_20	25	29450652	8.78E-05	147.6048595	37.63546448	0.070595797
113359_61	25	29490906	8.78E-05	147.6048595	37.63546448	0.070595797
114020_52	26	27655266	8.78E-05	147.6048595	37.63546448	0.070595797
114425_67	27	24432266	8.78E-05	147.6048595	37.63546448	0.070595797

116022_15	32	1157588	8.78E-05	147.6048595	37.63546448	0.070595797
11862_80	4	60816632	8.78E-05	147.6048595	37.63546448	0.070595797
122881_23	2	86010010	8.78E-05	147.6048595	37.63546448	0.070595797
123538_61	3	119174281	8.78E-05	147.6048595	37.63546448	0.070595797
126655_63	5	13546040	8.78E-05	73.80242973	18.81773224	0.070595797
128093_77	5	95444948	8.78E-05	147.6048595	37.63546448	0.070595797
132353_78	8	63682970	8.78E-05	147.6048595	37.63546448	0.070595797
132353_8	8	63682900	8.78E-05	147.6048595	37.63546448	0.070595797
14578_66	5	651718	8.78E-05	147.6048595	37.63546448	0.070595797
14827_24	5	7342003	8.78E-05	147.6048595	37.63546448	0.070595797
150408_45	18	587222	8.78E-05	147.6048595	37.63546448	0.070595797
151471_86	19	2797498	8.78E-05	147.6048595	37.63546448	0.070595797
156787_12	23	32892406	8.78E-05	147.6048595	37.63546448	0.070595797
156793_45	23	32932201	8.78E-05	147.6048595	37.63546448	0.070595797
157643_51	25	1009197	8.78E-05	147.6048595	37.63546448	0.070595797
163463_25	32	13225590	8.78E-05	73.80242973	18.81773224	0.070595797
165176_60	33	2101688	8.78E-05	147.6048595	37.63546448	0.070595797
167501_67	36	6767445	8.78E-05	147.6048595	37.63546448	0.070595797
183670_10	6	79204331	8.78E-05	73.80242973	18.81773224	0.070595797
18753_40	6	73270341	8.78E-05	147.6048595	37.63546448	0.070595797
18990_78	6	82950531	8.78E-05	147.6048595	37.63546448	0.070595797
18990_82	6	82950527	8.78E-05	147.6048595	37.63546448	0.070595797
2238_79	1	3043961	8.78E-05	147.6048595	37.63546448	0.070595797
24121_16	8	63692650	8.78E-05	147.6048595	37.63546448	0.070595797
24134_27	8	64506654	8.78E-05	147.6048595	37.63546448	0.070595797
24135_88	8	64536965	8.78E-05	147.6048595	37.63546448	0.070595797
2841_89	1	57116196	8.78E-05	147.6048595	37.63546448	0.070595797
30809_34	10	59936853	8.78E-05	147.6048595	37.63546448	0.070595797
30809_57	10	59936830	8.78E-05	147.6048595	37.63546448	0.070595797
30811_29	10	59942630	8.78E-05	147.6048595	37.63546448	0.070595797
34604_79	11	82887557	8.78E-05	147.6048595	37.63546448	0.070595797
34605_47	11	82894249	8.78E-05	147.6048595	37.63546448	0.070595797
34605_80	11	82894216	8.78E-05	147.6048595	37.63546448	0.070595797
34609_10	11	82917635	8.78E-05	73.80242973	18.81773224	0.070595797
36593_11	12	58430970	8.78E-05	147.6048595	37.63546448	0.070595797
36593_40	12	58430999	8.78E-05	147.6048595	37.63546448	0.070595797
4897_78	2	121947664	8.78E-05	147.6048595	37.63546448	0.070595797
49697_30	16	43307480	8.78E-05	147.6048595	37.63546448	0.070595797
58078_72	19	1797536	8.78E-05	147.6048595	37.63546448	0.070595797
58849_24	19	2803512	8.78E-05	147.6048595	37.63546448	0.070595797
59026_18	19	3237727	8.78E-05	147.6048595	37.63546448	0.070595797
60056_15	19	44952068	8.78E-05	147.6048595	37.63546448	0.070595797
61133_80	20	20204623	8.78E-05	147.6048595	37.63546448	0.070595797
61226_26	20	22061367	8.78E-05	147.6048595	37.63546448	0.070595797
66399_13	22	27823476	8.78E-05	147.6048595	37.63546448	0.070595797
6683_71	2	95522618	8.78E-05	147.6048595	37.63546448	0.070595797
68124_61	23	23312358	8.78E-05	147.6048595	37.63546448	0.070595797
68725_27	23	32895051	8.78E-05	147.6048595	37.63546448	0.070595797
68725_48	23	32895030	8.78E-05	147.6048595	37.63546448	0.070595797
69340_70	24	16246498	8.78E-05	147.6048595	37.63546448	0.070595797
69340_86	24	16246482	8.78E-05	147.6048595	37.63546448	0.070595797
70554_15	25	2812162	8.78E-05	147.6048595	37.63546448	0.070595797
70604_64	25	29272275	8.78E-05	147.6048595	37.63546448	0.070595797
70604_72	25	29272283	8.78E-05	147.6048595	37.63546448	0.070595797
70606_47	25	29299750	8.78E-05	147.6048595	37.63546448	0.070595797
70611_18	25	29409865	8.78E-05	147.6048595	37.63546448	0.070595797
70616_65	25	29477517	8.78E-05	147.6048595	37.63546448	0.070595797
70617_37	25	29486369	8.78E-05	147.6048595	37.63546448	0.070595797
70621_37	25	29533416	8.78E-05	147.6048595	37.63546448	0.070595797
72372_46	26	1837708	8.78E-05	147.6048595	37.63546448	0.070595797
73059_61	26	27742159	8.78E-05	73.80242973	18.81773224	0.070595797
75421_60	28	10578630	8.78E-05	147.6048595	37.63546448	0.070595797
76142_17	28	6462287	8.78E-05	147.6048595	37.63546448	0.070595797
77818_53	30	18915766	8.78E-05	147.6048595	37.63546448	0.070595797
81336_59	32	230582	8.78E-05	147.6048595	37.63546448	0.070595797
84081_54	33	6469430	8.78E-05	147.6048595	37.63546448	0.070595797
96792_91	4	53749956	8.78E-05	147.6048595	37.63546448	0.070595797
99001_39	6	91232606	8.78E-05	147.6048595	37.63546448	0.070595797
99001_41	6	91232604	8.78E-05	147.6048595	37.63546448	0.070595797

99001_65	6	91232580	8.78E-05	147.6048595	37.63546448	0.070595797
31711_25	11	22260198	9.18E-05	3.601049694	0.920672205	0.071714731
31711_44	11	22260217	9.18E-05	3.601049694	0.920672205	0.071714731
31711_76	11	22260249	9.18E-05	3.601049694	0.920672205	0.071714731
31711_86	11	22260259	9.18E-05	3.601049694	0.920672205	0.071714731
25407_22	9	13642210	0.000103001	3.546232786	0.913174724	0.076482823
75031_56	27	4273152	0.000103519	-13.03769634	3.358332745	0.076482823
105108_68	13	44647227	0.0001109	-40.18	10.39474275	0.076482823
105437_22	13	9050259	0.0001109	-40.18	10.39474275	0.076482823
148134_68	17	2587074	0.0001109	-40.18	10.39474275	0.076482823
15707_39	5	92600307	0.0001109	-40.18	10.39474275	0.076482823
16684_15	5	97329545	0.0001109	-40.18	10.39474275	0.076482823
22446_42	7	83006915	0.0001109	-40.18	10.39474275	0.076482823
22908_69	7	86007410	0.0001109	-40.18	10.39474275	0.076482823
22916_18	7	86030743	0.0001109	-40.18	10.39474275	0.076482823
23023_90	7	86459359	0.0001109	-40.18	10.39474275	0.076482823
35566_89	12	14779076	0.0001109	-40.18	10.39474275	0.076482823
38685_29	13	12770694	0.0001109	-40.18	10.39474275	0.076482823
39339_42	13	18420078	0.0001109	-40.18	10.39474275	0.076482823
40207_28	13	28587340	0.0001109	-40.18	10.39474275	0.076482823
42394_15	13	8748123	0.0001109	-40.18	10.39474275	0.076482823
42623_74	14	12783233	0.0001109	-20.09	5.197371377	0.076482823
79062_38	31	12919751	0.0001109	-40.18	10.39474275	0.076482823
8837_26	3	45326924	0.0001109	-40.18	10.39474275	0.076482823
82233_82	32	625797	0.000116528	-33.41666667	8.672181431	0.079871162
127385_39	5	73877642	0.000117603	61.66918413	16.01352219	0.080116462
79172_13	31	14169096	0.00012044	63.21098036	16.43880425	0.081551412
110358_10	19	43884181	0.000128437	-15.27192982	3.988036125	0.085925246
112264_38	22	3479140	0.000128437	-15.27192982	3.988036125	0.085925246
53052_79	17	31131176	0.000129989	-5.166665509	1.350240244	0.085987161
29880_61	10	3579814	0.000130069	87.4876112	22.86464034	0.085987161
153591_7	20	5788044	0.000139029	-21.98170732	5.769644742	0.091369907
5459_7	2	21281925	0.000141546	77.20195379	20.28719863	0.092479806
141357_76	13	33524311	0.000144613	43.7847629	11.52187536	0.093712372
59595_42	19	42118013	0.00014511	-15.61111111	4.108952136	0.093712372
86568_60	35	23506862	0.000148414	-3.971238938	1.046795903	0.09504903
111358_63	21	2020748	0.000148881	49.99067609	13.17996772	0.09504903
21775_61	7	68910611	0.00015224	-4.292745457	1.13343181	0.096640996
116246_71	32	20190978	0.000153758	-5.393797019	1.425075808	0.097053708
38715_9	13	12937053	0.000156122	40.05003549	10.59209881	0.097992048
62615_16	20	5746481	0.000166889	-17.73790323	4.711899298	0.098290528
62615_16	20	5746481	0.000166889	-17.73790323	4.711899298	0.098290528
62621_11	20	5815981	0.000166889	-17.73790323	4.711899298	0.098290528
59609_65	19	42166310	0.000167828	-18.25641026	4.851443921	0.098290528
111625_85	21	29971234	0.000168477	92.0539808	24.4686218	0.098290528
117567_73	35	11589395	0.000168477	92.0539808	24.4686218	0.098290528
49308_60	16	37428404	0.000168477	92.0539808	24.4686218	0.098290528
84712_59	34	17615157	0.000168477	92.0539808	24.4686218	0.098290528
84712_76	34	17615140	0.000168477	92.0539808	24.4686218	0.098290528
84716_28	34	17644716	0.000168477	92.0539808	24.4686218	0.098290528
9385_70	3	66424822	0.000168477	46.0269904	12.2343109	0.098290528
107371_57	16	38119791	0.000169794	103.7392582	27.58893907	0.098290528
146179_31	16	37840852	0.000169794	103.7392582	27.58893907	0.098290528
15631_24	5	92016813	0.000169794	103.7392582	27.58893907	0.098290528
34447_68	11	81918150	0.000169794	103.7392582	27.58893907	0.098290528
45801_76	15	2764397	0.000169794	103.7392582	27.58893907	0.098290528
8182_70	3	23158201	0.000170702	-18.83599089	5.011116006	0.098306707

Breed	Age	flank	feature	class	assembly	genomic_accession
Marecha	4	GGCCTACCGAAAAGGGGGGTACACCCACCTTGAAACCAGGAGGGTATGTCCGTGCCAACATGAAGGGAAT				
Marecha	2	ccttttgacacgTTCCTA	gene	protein_coding	GCF_000803125.2	NC_044531.1
Lassi	WeaningWt	GAGCTGGGTGCRTG	gene	protein_coding	GCF_000803125.2	NC_044528.1
Lassi	2	TGAGGGCCCTCGGG	gene	protein_coding	GCF_000803125.2	NC_044523.1
Lassi	2	GCTTGGACAAGCTGTGTGTGACCGGAGCTCAGGGACGCTGACCTGCCCCGTCCTgtctgtcctcagggagcagcC[T/				
Marecha	4	GCCCGGTGTCATAT	gene	protein_coding	GCF_000803125.2	NC_044512.1
Marecha	4	TGAGGAGACCAGA	gene	protein_coding	GCF_000803125.2	NC_044523.1
Marecha	4	GGAAATGATTTGA	gene	protein_coding	GCF_000803125.2	NC_044512.1
Lassi	BirthWt	TTCTTCTTTAGCATC	gene	protein_coding	GCF_000803125.2	NC_044544.1
Lassi	2	CTGCAGATTCCTTGGACCCCTTTGAGAATCTCGGAGAGCTATGAACTCTCACCTCCAAGAAGGCACATCCCA				
Lassi	1	TGAGGGCCCTCGGG	gene	protein_coding	GCF_000803125.2	NC_044523.1
Marecha	4	GAATATCCGGGTCTTTGATTCAATGAATCCATCTCTCCCACATTTCAAGGGCTTGGAGAGGGAACAGAGAG				
Marecha	4	CCTCCATGCGGTAGTCTGCTTTGCCCATGTGTCAAACAAGCCCRGACTTTGAATGTTCACCTTGACAGGGAAA				
Marecha	4	taaagaaattctcCAAA	gene	protein_coding	GCF_000803125.2	NC_044540.1
Marecha	4	CCCCAGGGTGTGCG	gene	protein_coding	GCF_000803125.2	NC_044511.1
Marecha	4	TCAATTCCTTTTTCC	gene	protein_coding	GCF_000803125.2	NC_044511.1
Marecha	4	GATCCCCCATCAGA	gene	protein_coding	GCF_000803125.2	NC_044528.1
Marecha	4	TGCAGAAACGTGGCGCCTGCGGAGAAGTCCGCAACCGACCGCGTGGCATTAGACGTGACTTTGTGCTGGTG				
Marecha	4	TTCTGGTGGTCTGTGTCTGCaccgggctggggctggggcagcggcAGCTCTCGGGTTCTGCTTTGGTTTATTACA[C/T]				
Marecha	4	AAAggatccccacccctcccgaCTGCAGAGTTCAGGCCCAGAATGGGTCCTCCCCAGCAGGGGGCAGTGTGGGT[G				
Marecha	4	ACGCACTGTGCAGCGTGTTTCATGAATGCTGCAGGGTTGACACCACGTTCTTCTGTCACTGAATTGCCCTGTG				
Marecha	4	CCCCGTAGGTGAGC	gene	protein_coding	GCF_000803125.2	NC_044521.1
Marecha	4	GCCAGAAGTGGTG	gene	protein_coding	GCF_000803125.2	NC_044516.1
Marecha	4	ACGCTTGCTGGGGAGAGCGCCAAGCCCTCCTCCAGGACAGGctgaccMcagccccMccccSacMgGCMGGCCC[				
Marecha	4	ACTACCCAAATTAT	gene	protein_coding	GCF_000803125.2	NC_044525.1
Marecha	4	ATGGTTGGTCATCT	gene	protein_coding	GCF_000803125.2	NC_044531.1
Marecha	4	CCCACCTGCACAAC	gene	protein_coding	GCF_000803125.2	NC_044531.1
Marecha	4	AAAACTGCAGCTGT	gene	protein_coding	GCF_000803125.2	NC_044531.1
Marecha	4	GCTAACACTGAGAA	gene	protein_coding	GCF_000803125.2	NC_044537.1
Marecha	4	GGAAACCACCCCCAGATCTGGAAGAAMATGAAGCAGGGATATGAAAGGTAGAAAAAGGAKGGAGGG				
Marecha	4	GCCGCGAGTCCGGC	gene	protein_coding	GCF_000803125.2	NC_044545.1
Lassi	2	AACTATTGGTATTTAAGCAAGCAGGTGGGTGAGTCTGCAGTTGTGTttggctttcagcttttctccagGTGCAAG[A/				
Marecha	2	TCTGGAAAAAACTGCTTCCCGGACTCATTTCATTAAAACATCTCAGTCCTTGggtttgaagttatttttaaagag[G/				
Marecha	2	AAGGCTGTTTCATG	gene	protein_coding	GCF_000803125.2	NC_044516.1
Marecha	2	CTCTGTACAGAGTGAAAGGAGGCCTCAGCTAATCAACAAAGAAATGTTCTAGCCATCTTTCAACTTTCAGAT				
Marecha	4	GGGGAGTGTTCCCTTCTCGGACATCACCTGAGGTCAGTCCTAGGAGAGGCCAGGGGACTGCAGCTCAGACT				
Marecha	4	TGCAGCTCAGACTGTGCKGGGGGCAAAGGAGGCAMTGGCTTGTCCTGRGCAGGGGTGCAGCTCAGCAGGG				
Marecha	4	AACAAGTGTGCACA	gene	protein_coding	GCF_000803125.2	NC_044543.1
Marecha	4	ACTGGGCTGACCTG	gene	protein_coding	GCF_000803125.2	NC_044543.1
Marecha	4	GGAGACCCCCACGA	gene	protein_coding	GCF_000803125.2	NC_044523.1
Marecha	4	GCAGAGTGCACTctgcagccagccagcctgcACATTCTCAGTACAACCAGCRGGTTCAGACAAAGTCCATGTGAT[T				
Marecha	4	GCAGCCAAGACCAG	gene	protein_coding	GCF_000803125.2	NC_044543.1
Marecha	4	CTCAGAGGCCAGCATGCTACCTGGGCTCTCCTGGAGCCAACCCCTTTCAGGATGACCTCAGCTGCAGGACACC				
Marecha	4	TGACCTCAGCTGCAGGACACCAASCCTTCTTCCCAGGAGAGGCCACCTCCCCAGGCAGGTCCCAGCAGCCAga				
Marecha	4	ACTGCAGGACCCCT	gene	protein_coding	GCF_000803125.2	NC_044519.1
Marecha	4	GAAaggagtggggtga	gene	protein_coding	GCF_000803125.2	NC_044522.1
Marecha	4	GAAaggagtggggtga	gene	protein_coding	GCF_000803125.2	NC_044522.1
Marecha	4	CCCTCCCGTCGGAGAACGCCTGGCAGCGGCGCCGGGAGGCCCCTTCTCTGCCGCCCCACAGAGCCTGCAGCAC				
Marecha	4	AGGAGTAGTGGTAAGCTGGGTTGAGTGAGGTTGAGAWTTCAgctaagaaaaagcaaaatagtgaCCACAGAAT				
Marecha	4	ACTACGCACTCCTGCAGGCCAGCTCTGCCACATCCCAGGGGGGCCCTAACTGCACCGGAGGCAGCATCAGAC				
Marecha	4	AATCAACATCTCTCCAGGCAGTAGCTTTTGGAGTCAACTTACTTTTTCCAaaatctgcttcctcctcctcaggga[T/C]G				
Marecha	4	ACACGTCAAATCCCACGCCTCGTTCTGCAGAAGTTGACCTCTATGAGGCAGTACTGGCCTCTGAGCTCAGGG				
Marecha	4	AGAAGTTGACCTCTATGAGGCAGTACTGGCCTCTGAGCTCAGGGTCTYCTGCCATGTGATGGCTCTGTCACTT				
Marecha	4	ACGTGACAGGCGGGGAGGGGCGGTCCTGGGTGCCGGTTCTTCGCTGCMCTCCTCAGGCCCCAGCGGCCCTTC				
Marecha	4	GTTCCCACTCGCCAGGCAGTCGGAGCCCACGTGACAGGCGGGGAGGGGCGGTCCTGGGTGCCGGTTCTTCGC				
Marecha	4	CCTCAATGGCCTGG	gene	protein_coding	GCF_000803125.2	NC_044526.1
Marecha	4	ggggcgcCAGCACCAAGGCCTCCAAGAGCGTTAGAACTCGTGGTCCAAAGCCCTGTCAAGGCTGCAGAGTTAA				
Marecha	4	gggctggcagggctggg	gene	protein_coding	GCF_000803125.2	NC_044527.1
Marecha	4	CACAGAGTGCTGTC	gene	protein_coding	GCF_000803125.2	NC_044528.1
Marecha	4	GGGAGACCCAGGC	gene	protein_coding	GCF_000803125.2	NC_044528.1
Marecha	4	CAGGTCTATTATAG	gene	protein_coding	GCF_000803125.2	NC_044528.1
Marecha	4	gagctgggtatttctcaa	gene	tRNA	GCF_000803125.2	NC_044530.1
Marecha	4	AAGCTGCCGCAGAT	gene	lncRNA	GCF_000803125.2	NC_044541.1
Marecha	4	CTGCCGCAGATGGC	gene	lncRNA	GCF_000803125.2	NC_044541.1
Marecha	4	GCAGATGGCgttact	gene	lncRNA	GCF_000803125.2	NC_044541.1
Marecha	4	CAATTTGTCCGTAAAGAAGagataatttttctctaatttacaCAAAGTTCCTTGCATACCTGCAGGGGCCCTGCC[A/G				
Lassi	WeaningWt	GTGGCAGAGCAGA	gene	protein_coding	GCF_000803125.2	NC_044513.1

Marecha		4	cttataatttaataaaagataaagtaaGGGCCCCAAATGGAGTAGGGTCCTTTCTCTGCAGTGCAGAGGAGATAG[T				
Marecha		4	GCAATCagccctcccc	gene	lncRNA	GCF_000803125.2	NC_044511.1
Lassi		1	AAATTCAGCAAACT	gene	protein_coding	GCF_000803125.2	NC_044520.1
Lassi		1	CTGCAGATTCCTTGGACCCCTTTGAGAATCTCGGAGAGCTATGAACTCTCACCTCCAAGAAGGCACATCCCAC				
Marecha		4	TACAGATGATTAGTTCAAGTATCCATAAAAGGAATCTGCAGCAATGTCAAGCGTTCATGGGAGAGGGAGA				
Marecha		4	CTGGGAGCCGTACCAGCAGTGAGGCAGGCCGTGACGCAGTAGCCTTGCCTGCGGCCGGCCCGCGTCAGGCCG				
Marecha		4	CCCAGCAGCATGGGCTCTGGTCAATTCARTTCTCAGACCACACAGGCCTGGGCTGTGCCTTCCCAGGAGGGA				
Marecha		4	AGCCTCAGAGGCTCTGCATGGGATGCTTTACAGATTAGATCCGGAAGCCCAGCAGCATGGGCTCTGGTCAAT				
Lassi	WeaningWt		AGGGGTCCTCTGAAACCGGGCAGGGTGCTATCCTGCCACACTCACTTCCCACCTCTGACCGTGCAAGGCAGG				
Lassi		2	gacccctctccccactgcaccCCCAACCTGGCTCCGCGCCTCTTTGGGCCGGGGGCCGGGGCTGGGCACTGGGCA[T/G				
Lassi		2	ACATGGCTRCCCCAGGGGGTCCTCTGCAAGCATCAGTCAGGTCCTGTAATCCCTGCGCTCACAACCACTCCCTG				
Lassi		2	GAGCGTGGCAAGT	gene	protein_coding	GCF_000803125.2	NC_044528.1
Lassi		2	ATCAAGTGAGGTCATTCAGAGCTGTCCCTGCAGATRTGGCCACACTGCTCCACCCTGCCCCCCCTAGAATTCAC				
Lassi		2	CAGTGTATATTCCTGTAGGCTGTAGTTTCCAAAGTCACAAATCAAGTGAGGTCATTCAGAGCTGTCCCTGCA				
Lassi	BirthWt		GCAGGCCACCAGG	gene	protein_coding	GCF_000803125.2	NC_044518.1
Marecha		4	GCATCTGTGTGCACACGGCTGTGTGCCCATGTGTGTGTTGGCTTCCATGTGCACATCACTGTGCTGCAGTACA				
Marecha		4	TCATCCCGCCCCACAMCTCTGCGGGCTGAAACCTGCGGCTTCTCAGGGCCCCGCAGCAACATCCCTGCTGCAC				
Marecha		4	CCAGTTTGCTTCCTGCAGCTGAGGACGCCCAGACCAAAATGAAAGCAAGCAACAGGGAAAGGAGCGCATG				
Lassi	BirthWt		TGCTGGATGGTGG	gene	protein_coding	GCF_000803125.2	NC_044538.1
Lassi	WeaningWt		TGGAGGCTGGAGACCTAACGCACCGCggtcctgcctcccagctgaCCTGTACACCCGGGGCACCAAGAAAGACGC[				
Marecha		4	ACAGCCGTCCGGGGGCAGCTCCCTGTCTTCCCTCCCGCTCGCTCACTCCTCTCTCGCTAGGGACAGGAGCCAGG				
Marecha		4	CRGACTTTGAATGTTCACCTTGACAGGGAAAAGYGGGAACAGGACACATGgataaggagaaatgaaaacttggg				
Marecha		4	TTCCAGCTCGTCTGCAGCCACGCTGGTGGCCTCCTATGTTGTTCTGCCAATTCCACCCCCcSMcYccYtYcttcc[T/				
Marecha		4	agcCTTCCAGCATGT	gene	protein_coding	GCF_000803125.2	NC_044542.1
Lassi		2	CGTGGCTTTGTGAG	gene	protein_coding	GCF_000803125.2	NC_044521.1
Lassi	WeaningWt		GACCTGTTCATTTT	gene	protein_coding	GCF_000803125.2	NC_044542.1
Marecha		4	AGTTAAAAGAATC	gene	protein_coding	GCF_000803125.2	NC_044517.1
Marecha		4	GCTTGGGAGTCAGA	gene	protein_coding	GCF_000803125.2	NC_044528.1
Lassi		2	TGCATGGTGATGTCTGAGCAAACACTTGCTGAACGAGTGAGCCCAGAATGGGCCAGTCGAGCTGTTAGGAC				
Lassi		1	CGGGCTCCAAAGAGCAAGGTGGCACKGCMAGAAGKGACMAAGRGMAGGTGAGTCKGGAGAGGGATGT				
Lassi	WeaningWt		CTTGTCTTTAAAGA	gene	protein_coding	GCF_000803125.2	NC_044531.1
Marecha	BirthWt		CTGGGACAGAGGT	gene	protein_coding	GCF_000803125.2	NC_044519.1
Marecha		4	aggGAGACAGGAA	gene	protein_coding	GCF_000803125.2	NC_044512.1
Lassi	WeaningWt		aaaatgtgaaaaaccgTCGCTGCAGGCTTAGGAGAATGccaggaagaaagacagagaaattgTGTTTCTAAAACC[A/G				
Marecha		4	CCTGGCAGAGAGATGGAACCAAGACGGAGGTGAGGCGTCCAGGGCCAGATGACCCGGGCAGTGGAGCTG				
Marecha		4	GGCCTGCCTGCAGG	gene	lncRNA	GCF_000803125.2	NC_044521.1
Marecha		2	CTCCACCACCAGAG	gene	protein_coding	GCF_000803125.2	NC_044538.1
Marecha		4	GAAGGGAGCTCAGCAAACAGGCAGCGCCCGCCTGGCTCAGCGCTGGGTAAACAGGACTCTGCCCYGCACCT				
Marecha		2	GCTGCAGGTCCCCT	gene	protein_coding	GCF_000803125.2	NC_044543.1
Marecha		4	AACTGGtcacccttccc	gene	lncRNA	GCF_000803125.2	NC_044522.1
Marecha		4	AGGTGTGCAAGCC	gene	protein_coding	GCF_000803125.2	NC_044522.1
Marecha		4	GGCTGCTGTAGCCCGAGTTACTGGTTGCAAGTAGCAGAAACCGACTCTGGTCACTGAAGCGGGAAAGAATT				
Lassi		2	gcgcccGGCGGGGCGAGGGAGCAGGAGGCTCTGAGCTGCAGACGCGGGCCCCGAAGCCGCCTGTCGTCGCGG				
Lassi		2	GGCTGGCCTCTGGG	gene	protein_coding	GCF_000803125.2	NC_044522.1
Lassi		2	actccctgccTCTAAGAAATCCCCTTCTTTGGTGTGGCMTGTCTCATCTCAGGAGAATTACAGCTGGGAAGGTG				
Lassi		2	CAGGACTTAGATGATTAGATTCTTTCAGGGAGGCGTCCAGCTGGGACATCTCTGCAGAAGTGAAACTTTGC				
Lassi		2	ATGATTAGATTCTTTCAGGGAGGCGTCCAGCTGGGACATCTCTGCAGAAGTGAAACTTTGCTAACRTAGGA				
Marecha		2	CTTGCTGGAGACAA	gene	protein_coding	GCF_000803125.2	NC_044514.1
Marecha		4	CGTCTGGCCCTGGG	gene	lncRNA	GCF_000803125.2	NC_044524.1
Lassi	BirthWt		gggaggaaggtaattaggtctgtttatgtatttattgaaacagagggactggggattgaacccgggaccttgtgc[G/A]tgctaagcac				
Lassi		2	ACCATCCCCTAGGTAGCTGAGAATTCTCTACTGGATATTCTGCAGTTGACCAAACAcgaggggaagaaagaggca				
Lassi		1	TTTATCTTGTCCTTCACTCAGTCACTCCTTCRTTTATTtacacaacWaatatttactgagtgcctgagGTCCAAG[A/G]CT				
Lassi	BirthWt		TGATTAAACCGCGT	gene	protein_coding	GCF_000803125.2	NC_044543.1
Marecha		4	TGGAGGGAGCATG	gene	protein_coding	GCF_000803125.2	NC_044537.1
Marecha		4	TCCAGACATTGCACGGCCCCCAGAACCCCACACGGTGACTTCCAGCCACTTACAGAAAATGTCCCCAAGCCCA				
Marecha		4	ATCTTGATGGCTGC	gene	lncRNA	GCF_000803125.2	NC_044521.1
Marecha		4	CCTGGCCATAGAGT	gene	protein_coding	GCF_000803125.2	NC_044530.1
Marecha		4	TGCTCTGGCCTGCGGCGGGTGGGGGCAAGGTTATTGGCCTCCAGggtcagaagaaacaaaaaaacttcgGCGGG[				
Marecha		4	CTGCAGGCCGGGCGGGGACCGCCGAGGCTCCCGCCTGGGAGCCGGGGACGCATCTCAGCTCCCTgcagccttccc				
Marecha		4	CTGCTGTGGCCCCY	gene	protein_coding	GCF_000803125.2	NC_044523.1
Marecha		4	CAGCTtgtcccagctccttccctggtTTCTTTGCCCCAATTCCCCTCTCTTGGACTGAGGGGTCCCAGAGAATGT[A/G]				
Marecha		4	GGCCCGCCAGCCGA	gene	protein_coding	GCF_000803125.2	NC_044526.1
Marecha		4	ACCAGAGGCGTAGAATCTCACATCTTCCCGGCACCGTAGTCTCGGGAAGGCCAAYGGAGATTACACRACTTC				
Marecha		4	tctgcagggCAAAAGGGCTGAGGCAGGAGCCTCTCCTGGCAGTTATCTGACTTTCTTCTTGGGGAGATGTTTGC				
Marecha		4	GAGGGGGACCGGG	gene	protein_coding	GCF_000803125.2	NC_044524.1
Lassi	WeaningWt		GTGTGTAGTGCACACTGTCATACACCTGCCGGTGCCTACACTGCTGGTGCCCTCAGACTCCAGCTGCAGTGAG				
Marecha		4	GAGCCTGCCATGTG	gene	protein_coding	GCF_000803125.2	NC_044523.1
Lassi	BirthWt		CGCCTGCTTCTGAC	gene	protein_coding	GCF_000803125.2	NC_044532.1
Lassi		1	ACCATCCCCTAGGTAGCTGAGAATTCTCTACTGGATATTCTGCAGTTGACCAAACAcgaggggaagaaagaggca				

Marecha		4	GAGGTCTCTGCAGG	gene	protein_coding	GCF_000803125.2	NC_044532.1
Marecha	BirthWt		GATTCTCGTGTGAG	gene	protein_coding	GCF_000803125.2	NC_044522.1
Lassi	BirthWt		CTTTGTCAGTCTGA	gene	protein_coding	GCF_000803125.2	NC_044514.1
Lassi	BirthWt		TATCTGTTGCTCCA	gene	protein_coding	GCF_000803125.2	NC_044514.1
Lassi	BirthWt		GCTGGCCTCGTCCC	gene	protein_coding	GCF_000803125.2	NC_044514.1
Lassi	BirthWt		CGGCAGGACGTGG	gene	protein_coding	GCF_000803125.2	NC_044514.1
Marecha		4	AGGCGGGCTGGGC	gene	protein_coding	GCF_000803125.2	NC_044522.1
Marecha		4	gGGGCCAGGTCTGG	gene	protein_coding	GCF_000803125.2	NC_044530.1
Marecha		4	AAGAGGAGTGGCAGGGTGGGAGGCCCCAAGGGAACAGAAGAGGGGAAACTGCAGCARTAGRGATTGAG				
Lassi	BirthWt		GCCGACCCCGCTCCGGGACGTGAGCATGCAGGAAACTTCTTCCTCCCACACCTCACTCCYGTTTCCTGCCGTGG				
Lassi		2	GGGCCTCCCAGTGA	gene	protein_coding	GCF_000803125.2	NC_044532.1
Lassi		2	CTCAGGCACTGTGG	gene	protein_coding	GCF_000803125.2	NC_044532.1
Lassi		1	gcgcccGGCGGGGCGAGGGAGCAGGAGGCTCTGAGCTGCAGACGCGGGCCCCGAAGCCGCCTGTCGTCGCGG				
Lassi		1	GGCTGGCCTCTGGG	gene	protein_coding	GCF_000803125.2	NC_044522.1
Lassi		1	CAGGACTTAGATGATTAGATTCTTTCAGGGAGGCGTCCAGCTGGGACATCTCTGCAGAAGTGAAACTTTGC				
Lassi		1	ATGATTAGATTCTTTCAGGGAGGCGTCCAGCTGGGACATCTCTGCAGAAGTGAAACTTTGCTAACRTAGGA				
Marecha		4	AAAGCAGGCTAGG	gene	protein_coding	GCF_000803125.2	NC_044526.1
Marecha		4	ACTAATGCTGGGATGTTCAGACACCCTGTGGTGGGAAGGAAGGCCTTTTGGTGCCTCCATTAACTCCCTGCA				
Lassi		2	ttttctgttctttttctaa	gene	protein_coding	GCF_000803125.2	NC_044527.1
Marecha	BirthWt		attttgttttaataataa	gene	protein_coding	GCF_000803125.2	NC_044526.1
Lassi	WeaningWt		GGTGTTTCCAGCTC	gene	protein_coding	GCF_000803125.2	NC_044524.1
Marecha	BirthWt		ATATTCCCCTTTCTC	gene	protein_coding	GCF_000803125.2	NC_044540.1
Lassi	WeaningWt		gccccggtggaagccgc	gene	protein_coding	GCF_000803125.2	NC_044530.1
Marecha		4	GAAACCCTCGGCCTCACCCTGCCCCAGCCGGGGGGCCTGTGCGTGTTTGAGTGCGTGCGTGAGTGCATGCGC				
Marecha		4	TTCACCAGGAGCTCTGGAAACTGCAGATCTATAGCAAGCAAACTGACGTTCGAGTTTTAAAATCCacaaaaa				
Marecha		4	ACAAATGASATGG	gene	protein_coding	GCF_000803125.2	NC_044536.1
Marecha		4	CCAGCAGCGGGGG	gene	protein_coding	GCF_000803125.2	NC_044546.1
Marecha		4	gaaaaacactgaggaCCAAAAAAATACATCTGGAAGCTTAATGTGGCAGATTGTAAGCTCTGCTGCAGACTCA				
Marecha		4	gTGGCAGAGGAGGCGCGGTCTTGACGGAGAGGCCCTGCAGAAAGTGCCACGGCCTCCACGGCCACCTCTTC				
Lassi	BirthWt		ATCCCTCCTTTGCA	gene	protein_coding	GCF_000803125.2	NC_044532.1
Lassi	WeaningWt		AGTGACTGCACTCT	gene	protein_coding	GCF_000803125.2	NC_044521.1
Lassi	WeaningWt		TTAGGGCAGATGG	gene	protein_coding	GCF_000803125.2	NC_044531.1
Marecha		4	TTGTCAAACAAAGA	gene	protein_coding	GCF_000803125.2	NC_044530.1
Marecha		4	TTGTCAAACAAAGA	gene	pseudogene	GCF_000803125.2	NC_044530.1
Marecha		4	cggggagggggcggag	gene	protein_coding	GCF_000803125.2	NC_044517.1
Marecha	BirthWt		GAGCCCCTCTCAGAGAAGGAGACCGCACTCGCTCGGTGCTAACTGGGCTGCCTGCGGTGTTCAAGGAAAAC				
Marecha		4	aaataaggaaaggGA	gene	protein_coding	GCF_000803125.2	NC_044541.1
Marecha		4	GAGAGCTTTGCAGT	gene	protein_coding	GCF_000803125.2	NC_044528.1
Marecha		4	ACAGAGGGGGCAG	gene	protein_coding	GCF_000803125.2	NC_044527.1
Marecha		4	ccagagccctgcagggtAGGGCTCATCCTTCCACYTCCAGGAGGTGATTCCTGCTCCCATGGGGTCCCAGCCACA[C/				
Lassi	BirthWt		accaggaagcagagaggccagCAAGGAGGTTCCTCCTGCAGGAACCAGGTCCATCCTTCTGGTTCACCAGGCCTC[C				
Lassi	WeaningWt		CACCGGCGCCCTGA	gene	protein_coding	GCF_000803125.2	NC_044531.1
Marecha		4	CCTGCAGGCACTGGACTGGCAGCCGCCTGGCGGCCACCACGGCAAGAACACCCGCCTCACGTGCGTGACGCA				
Marecha		4	TCAGCGGCTGCAGA	gene	protein_coding	GCF_000803125.2	NC_044524.1
Marecha		4	AGAAMGGctgaaMagtttctaaaattgGCCAGAGACGTGGATCCAGCTGCGGAGGACTCAGGATGAAGACAAG				
Marecha		4	CCTTACTCAGAGCA	gene	protein_coding	GCF_000803125.2	NC_044531.1
Marecha		4	AGTCTCGGGAAGGCCAAYGGAGATTACACRACTTCACTRGGCGATTCATCCGAACAGAGGACTATTGTCGA				
Marecha		4	TTAAAGGATACCAGAGGCGTAGAATCTCACATCTTCCCGGCACCGTAGTCTCGGGAAGGCCAAYGGAGATT				
Marecha		4	TTGGACTTGACATTAAAGGATACCAGAGGCGTAGAATCTCACATCTTCCCGGCACCGTAGTCTCGGGAAGG				
Marecha		4	CAGCTCTGTCACTG	gene	protein_coding	GCF_000803125.2	NC_044521.1
Lassi		1	AAAATGATCAGAGGTGGCGTCGTGGGTGTGTCCCCCAGCACTGCAGTTGCTGGTGTATCATGACGATTCACT				
Marecha		4	GGCGACTCGGAGC	gene	protein_coding	GCF_000803125.2	NC_044519.1
Marecha		4	CAACTaatgagaggaa	gene	protein_coding	GCF_000803125.2	NC_044541.1
Marecha		4	TGCAGCATCTCCAGCACTCCCGAACCTGAGTTCCAGCAGGTCTAAATGCATAATTCCTGTCCAAACAGGGAG				
Marecha		4	GAGGCCACAGGTGACCGAGGGTGCCGGGGCTTGTAGGCATTCTGAATCCTTACCCTGACGGCCTCCTTTCCA				
Lassi	BirthWt		TTATGAGCCGGTTC	gene	lncRNA	GCF_000803125.2	NC_044523.1
Lassi	BirthWt		AGAGGTCACGGGA	gene	protein_coding	GCF_000803125.2	NC_044514.1
Lassi	BirthWt		GCTTGACTTTTCAGAGAAGTACAAGATGTAATTTTAGGTTACAAGGATGCTGGAAGATGCAGAGTCTACTG				
Marecha		4	AGCATCCTTGGGGAGACCGCAGAGGCAAACGCAGGGGAGGTGCTCGAGGACACGCGCCACACAGCTGAGA				
Marecha		4	tgcagctgctcctgcCGC	gene	protein_coding	GCF_000803125.2	NC_044519.1
Lassi	WeaningWt		GCGCCCACCAGGAC	gene	protein_coding	GCF_000803125.2	NC_044530.1
Lassi		2	CTGGCGCCAAAGGCTGCAGTCATCCGCACTCCCCTCCCTGGTACCCACAAACGCTTTGctcacccaccccMgcc[T/				
Lassi		2	GAGTCCGGGGCCGC	gene	protein_coding	GCF_000803125.2	NC_044523.1
Lassi		2	CAGCGGGGTCTCTG	gene	protein_coding	GCF_000803125.2	NC_044523.1
Lassi		2	GAAACTGGGATTGTGACTGCAGTCAGTTGCTCTCGGGCTGAGATATTTGGACATTAGGGTTTGGAATGCAC				
Lassi		2	ATGGGCACAAGGAGAGAACCACCACTTAATTTCCAGCTTACTACACGAAGCATTCTGCAGGAGCACGGGAC				
Lassi		2	GAATCGGCACTGTTCCCAGTGGTGCTCGCCTGTATAGCTGAAAGAACACAGGTTTTGGGGCTGAATCTTCTA				
Lassi		2	GTGCAGGTGTTACTGCTTACAGAGCTCATGTACGCAGCAAGGGAGAAACCAGGAGGCAGAGCAACTATAA				
Lassi		2	GTCTGGTCTGCAGC	gene	protein_coding	GCF_000803125.2	NC_044537.1

Lassi	2	TGCTRTTCTCAGCTCTTCCCCTGCTCGAGCATCCTCACRCGTTCCCCTGGCTTCAGTTTTGAACACGTGCCTGCT[				
Lassi	2	GAGACTGGAAGCC	gene	protein_coding	GCF_000803125.2	NC_044514.1
Lassi	2	TTATTGAGAGCCTCCCACGGACCAGAAACTACAAAAATCAATCAACCCCTCCTGCAGCCATCCAGGAACTTA				
Lassi	2	GCTCTAGCATCAGCTGCAGATTTAGAACCACCTCCCAAGGTAATTACAGACGTAGGATCCACACTAAGTTCT				
Lassi	2	AACTTGCACACCTG	gene	protein_coding	GCF_000803125.2	NC_044515.1
Lassi	2	TGAGCTCAGTGTGTCCGGCTGGGCCCTGAGAACGAGGTGGCCTTGACCAGAGCCTGGGCTGSGGCCCKGGG				
Lassi	2	AGGACWCACACACTTGCSCTTGCCAATCTAGCACCACAGTTTATTCACAGGCAAGTAAATCAGGACTTCTGC				
Lassi	2	TTGTCTTCTTATTCCTATTCATCTCGACCAGTCCTGACCAACAGGTGCCTCCAAACACGTCAGCCTCTGCAGGA				
Lassi	2	CAGTCAGCCTGCAG	gene	protein_coding	GCF_000803125.2	NC_044515.1
Lassi	2	TTTCAAGAAGTCCC	gene	protein_coding	GCF_000803125.2	NC_044515.1
Lassi	2	GTGGCAAGCTCAGC	gene	protein_coding	GCF_000803125.2	NC_044528.1
Lassi	2	CTCCTGCTCCCCAGCTCGCCTGGTCCCCAGGCTCWGCGTGTGCGGCCGCAGCCACGGTCCCCTCAGCCCCTCA				
Lassi	2	CGTCGTGTCTGGTG	gene	protein_coding	GCF_000803125.2	NC_044533.1
Lassi	2	cctctgctcttgCCTGAC	gene	protein_coding	GCF_000803125.2	NC_044533.1
Lassi	2	ATCTCCCTTCCTGCC	gene	pseudogene	GCF_000803125.2	NC_044535.1
Lassi	2	TCCTCCCGCACCCCGGTCTCTCTTTCTGACAGatgaatcacctgggaatcttgttaaaatgcaggtctTGAATGG[G/A]CA				
Lassi	2	CAGAGCGGACAGG	gene	protein_coding	GCF_000803125.2	NC_044543.1
Lassi	2	CACTGAACTGCAGT	gene	lncRNA	GCF_000803125.2	NC_044546.1
Lassi	2	AAGTGAGCAGAAGGCAGCAGCCAGAGCCAGGCAGCAGATTGGGGTCCCAGCTAAATAGTCTGAGCTGCAG				
Lassi	2	TCCTCCACCCCCGGC	gene	protein_coding	GCF_000803125.2	NC_044516.1
Lassi	2	CGTGTGCAGGCAGTTCCGGGGGAAGAGGGGCTCTTCAGAGAGCCGTGGGTGTGCCCTGAGTCTACTCTCCCR				
Lassi	2	ACATCGTGTGCAGGCAGTTCCGGGGGAAGAGGGGCTCTTCAGAGAGCCGTGGGTGTGCCCTGAGTCTACTC				
Lassi	2	GCTGACAGCCCCCT	gene	protein_coding	GCF_000803125.2	NC_044511.1
Lassi	2	TATACACCACCATGCTGGTTTCAAACCGCATGATAGCTCTGAGACAAATACCAAGATTCTGCAGCACGTGAA				
Lassi	2	TGTGAATGCGGTCTGAGTGCCTCTGTCATCTCTGGATTTCATCCAAACCCTTCTATGACTTTAGATGCGGAGG				
Lassi	2	CTCCCATCCTAAGTCTCAAGTTCCCATTCTCAGAGTAAGGGTGGTATTGGTTTAGTTGGTGGGACAATCACCT				
Lassi	2	GGGACTGGAAAGC	gene	protein_coding	GCF_000803125.2	NC_044511.1
Lassi	2	AATGCCTGTGCAAA	gene	protein_coding	GCF_000803125.2	NC_044520.1
Lassi	2	TCACCGGGAGCACC	gene	protein_coding	GCF_000803125.2	NC_044520.1
Lassi	2	TCCACCTGACACCT	gene	protein_coding	GCF_000803125.2	NC_044520.1
Lassi	2	CTGACTGCCTGCGAAGTGCTTGCTCTCAGCCGGGATTGCACGAGCAGAAGCATTTATACTTTGgctgttttcttt[				
Lassi	2	CGGGCCCCATCTCCAGAGGCTAATTGTTTTCAGACAAGCCTGYTGGCAGGGGCTGAGTCCCCTCCCCTGCACA				
Lassi	2	GGAGCGCAGTCACCACCTGCACCTGCTGGGGCCCGGGCCCCATCTCCAGAGGCTAATTGTTTTCAGACAAGC				
Lassi	2	AGTCTAGGGAGGTCTGGGTGAGCGCAGACAGGTCTGTTTTGGGCAGYGAGGCTGTCCTTTGCCCCGTGGGA				
Lassi	2	cgccctcccccctcctctctcagagTATCAGCCAGCATTTGGGGTCACTAATGCACGTGAGGCTCTGCAGAGCACA[T/C]				
Lassi	2	GCCAGCATTTGGGGTCACTAATGCACGTGAGGCTCTGCAGAGCACAYGTGTCAACCCAGGCTGTGCTTGCAC				
Lassi	2	AGAGCAACAGGAT	gene	protein_coding	GCF_000803125.2	NC_044512.1
Lassi	2	AACCAGTGCAGGGAGATGAAGTCACTTGGTGGGTTCTGAGCTCcctgcaggaagccttccctccccccaggcctG[T/G]				
Lassi	2	GCAGCGGGATCAGC	gene	protein_coding	GCF_000803125.2	NC_044529.1
Lassi	2	CTCTCAGAGAAGGAGACCGCACTCGCTCGGTGCTAACTGGGCTGCCTGCGGTGTTCAAGGAAAACAGACRC				
Lassi	2	GCCCGTGATCGCTG	gene	protein_coding	GCF_000803125.2	NC_044529.1
Lassi	2	TGGGGAGAAGGGGTAGCTGGTCTTGACAGTGACAATGGCAGAGCAGCATGAGGATCGCTCTGCAGGGGT				
Lassi	2	CGGGGAAATGTTAAATCAGCGAATTGAGATGCCAGAGACGAAAGTGGGACTGCCACGTGGAGCCTGCCAC				
Lassi	2	ATGTTTTCTGGCTGC	gene	protein_coding	GCF_000803125.2	NC_044530.1
Lassi	2	CGTGACGCCGGCTC	gene	protein_coding	GCF_000803125.2	NC_044532.1
Lassi	2	CCCCTGCAGGCTAGAGGATATTGCAGGCGAGGACCCCAGAGCAGTCTCTGAACattcctgccccccacccccctSc[A/				
Lassi	2	GTGCCCCTAAGATCTGCAGGTCTCAAGAGTTAGGCAAAGGGGGTTTTCCTTCTTAGAGAGTCATAACAGGG				
Lassi	2	cttcctgcctcagtttcttc	gene	protein_coding	GCF_000803125.2	NC_044533.1
Lassi	2	GGAGtttcttaaaattc	gene	protein_coding	GCF_000803125.2	NC_044533.1
Lassi	2	AAAGGTGGGTGGGCTGAGTCCACGGAGCCCCCGGGCTCCAAAGAGCAAGGTGGCACKGCMAGAAGKGAC				
Lassi	2	GGCCCGGAGAAGGCTCAAAGGTGGGTGGGCTGAGTCCACGGAGCCCCCGGGCTCCAAAGAGCAAGGTGGC				
Lassi	2	CCGGGGCTGAGATG	gene	pseudogene	GCF_000803125.2	NC_044535.1
Lassi	2	gctcctcctcctgcaggtcccagcccagccttcctctcctccacgAAGCTTCTCTGGCCCTGCAACCCCTGCCTC[A/G]CAGTCTG				
Lassi	2	tcctgcaggtcccagcccagccttcctctcctccacgAAGCTTCTCTGGCCCTGCAACCCCTGCCTCRCAGTCTG[T/C]GTTGG				
Lassi	2	TTCATCTGAAGGGTCTACCTGGGGCCTCTGCAGCACAGAGTTCTCAGGTAGACACACTTCTTACCTGGCCACG				
Lassi	2	GGGACACAGCAGGGACCCCTCCTGCTCTcgagagaaggaagaacaaagatgTTCCCCTTTCTGGAAATTTGATAC[G				
Lassi	2	CCTCCTATAAGCAC	gene	lncRNA	GCF_000803125.2	NC_044535.1
Lassi	2	TCATCACTTTCCTTCTTGCCACAGATCCTTGGTTGCTCAATCGGCACTTAATCACTCAGTCACTTAAGGGTGCA				
Lassi	2	GTGGCCAGTCAAATTGACACATGGAATTAACCGTCACAACACCTCCTGATTACACAGAAGGTGCCAGTCCCT				
Lassi	2	TGGTTCTggcggtagagcacgtgctcagctgCAGGTGCCCCCCTCCAGCTGGGAGccccccMccMgccYcYcccc[A/C]gcA				
Lassi	2	CAAGCTCAGGGCAT	gene	protein_coding	GCF_000803125.2	NC_044536.1
Lassi	2	ccccaggggcctCTGGTGGGCCGGCTTCATGTGTTGTGTCTGGAAGGTCTCGGGCCTCAGGGCTGTGGTTGCCAC				
Lassi	2	GCTTCCTTGGCATGC	gene	protein_coding	GCF_000803125.2	NC_044538.1
Lassi	2	TATCCTGACATGTCC	gene	protein_coding	GCF_000803125.2	NC_044540.1
Lassi	2	TTACTCCCTCAGCCT	gene	protein_coding	GCF_000803125.2	NC_044542.1
Lassi	2	ACGTAGAAATCACG	gene	protein_coding	GCF_000803125.2	NC_044543.1
Lassi	2	GATAMGAGTTTTGGTCTTAGAACCAAGGAGTCTGAGTGCCAAGCCAGCTTTGCTACCAAATCTCTGTTAAG				
Lassi	2	ctattgcAACATGGGGTGACAGCGACACCGGCCTCGTATGGCCCTGAGGRGGGGGCAGTGCCTGGGCAGACTC				
Lassi	2	ccctattgcAACATGGGGTGACAGCGACACCGGCCTCGTATGGCCCTGAGGRGGGGGCAGTGCCTGGGCAGAC				

Lassi	2	GTGACCCtcgctaagcctcagtttccctattgcAACATGGGGTGACAGCGACACCGGCCTCGTATGGCCCTGAGG[A/G				
Lassi	BirthWt	GCAGCTCGGTGGG	gene	protein_coding	GCF_000803125.2	NC_044521.1
Lassi	BirthWt	GCGCGGGGGGTCCC	gene	protein_coding	GCF_000803125.2	NC_044521.1
Lassi	BirthWt	GCAGGGCTTATGCC	gene	protein_coding	GCF_000803125.2	NC_044521.1
Lassi	BirthWt	TGCCTCTCTGGTGCK	gene	protein_coding	GCF_000803125.2	NC_044521.1
Lassi	BirthWt	CCTGGGTTAGAGAGCAATTAACGTTAGTCCTCAGTTGCACGKGTTGGGCTCAGCTTTGGGACCCTCTGTGAA				
Lassi	WeaningWt	TGCCTTGACCCATCAGACCTGCAGCCGCTTGCAGCATCCCCTCTGCCCCTAGAGGGACTCCTGCTAAAAGCTGT				
Lassi	WeaningWt	ATATTGCTGCAGTTCATTAGAGACCAGAGATGGGCTCTAACTCTCCAACCAAGGCAGTCGGAGAGGTCTTGC				
Lassi	WeaningWt	GACCATCTGTATCCCTTGGCCTCTGCCCAGTTTTCAAGCCCAATTTTCAGTCTGCCAGGCCATTCTGAGCTCCCA				
Lassi	WeaningWt	TGTAATCTGCAGAG	gene	protein_coding	GCF_000803125.2	NC_044527.1
Lassi	WeaningWt	ACTGGACGGTCTTAATTCACTAGATtagattctctcttcctgtgtttgtattttccctctttttcccttggt[G/T]CCCCTTCTG				
Lassi	WeaningWt	GCGCGCAGCGCTGG	gene	protein_coding	GCF_000803125.2	NC_044515.1
Lassi	WeaningWt	GGAGCCCGGGAGGCCAGTCTCGCCTCTGAGTCTAAAAAGCTCTGCCAACTTAGGAAGGAAACTTGAAATTT				
Lassi	WeaningWt	CTCGCAGGGACAgg	gene	protein_coding	GCF_000803125.2	NC_044517.1
Lassi	WeaningWt	GAAGCTCCGTGACG	gene	protein_coding	GCF_000803125.2	NC_044517.1
Lassi	WeaningWt	TTGAGCCAAGACGC	gene	protein_coding	GCF_000803125.2	NC_044517.1
Lassi	WeaningWt	TGTGTGAAGGCACT	gene	protein_coding	GCF_000803125.2	NC_044522.1
Lassi	WeaningWt	ATGGTCTCTATTTAT	gene	protein_coding	GCF_000803125.2	NC_044523.1
Lassi	WeaningWt	CAGGTCCCTCTGCCATGTGTTCCCACAGCACGGCCTCTCCTTGGCCTCTGGACTCGTCCAGTCTCGGGCCTGGG				
Lassi	WeaningWt	TTATGTGTCCTCACTCACGTCAACGAGGGTGCTAGAACCCACCCCCAGACCAAGTGGGTCCCACAGCTCCCTT				
Lassi	WeaningWt	GAGCCTGCCATGTG	gene	protein_coding	GCF_000803125.2	NC_044523.1
Lassi	WeaningWt	CTGCAGACAGttgaggagggaaggcaggcgaATCCAGCGGAGAAGCAGTCCCCGTGGACCTCCTGTGTTCCTGTG[				
Lassi	WeaningWt	ATCAGGGAGCGTCT	gene	protein_coding	GCF_000803125.2	NC_044541.1
Lassi	WeaningWt	catccgGATAGAAGGCGGGGTTGTAACGCTGACTTGCCTCTTCAGTTTCCTGGGCTCtgacactcccctcccca[G/C]c				
Lassi	WeaningWt	AGCATGAATCTGTG	gene	protein_coding	GCF_000803125.2	NC_044542.1
Lassi	2	CACAGAGAGTGTCC	gene	protein_coding	GCF_000803125.2	NC_044515.1
Lassi	2	ctcgcCTCCCACTCTGT	gene	protein_coding	GCF_000803125.2	NC_044541.1
Lassi	WeaningWt	TGTGAAATTACATGGTCCATTAGAGACCAAAYCCAGTGCAGATGTGTGTGATTTCCGCTTTGCAGGWAATG				
Lassi	WeaningWt	CCCCCTCAACGCCAG	gene	protein_coding	GCF_000803125.2	NC_044532.1
Lassi	BirthWt	AGAGGCTGTCCTAA	gene	protein_coding	GCF_000803125.2	NC_044527.1
Lassi	2	AAATTCAGCAAACT	gene	protein_coding	GCF_000803125.2	NC_044520.1
Lassi	WeaningWt	ttcaaaaaatatttttctc	gene	protein_coding	GCF_000803125.2	NC_044530.1
Lassi	2	AGGCACCTGCTGACCGAGCGTCAGGTCCTCTGCGTGGCGTTGCCATCACCCCATCATAAATGGAAAGGGAGA				
Lassi	1	GTTACGCCAAGAtga	gene	protein_coding	GCF_000803125.2	NC_044523.1
Lassi	WeaningWt	TCTGCCCAGAGTcag	gene	protein_coding	GCF_000803125.2	NC_044529.1
Lassi	BirthWt	GATGATGAcggtggctgcagggccctgcctGAGTTAAAGGAGGGGCATCggaggtgggggcagctggaAGGGGTC[C/T]				
Lassi	2	AGTTAGGGAACCTG	gene	protein_coding	GCF_000803125.2	NC_044531.1
Lassi	BirthWt	GGTTCGAGGCTGTC	gene	protein_coding	GCF_000803125.2	NC_044517.1
Lassi	BirthWt	GATCTGCAGAATTG	gene	protein_coding	GCF_000803125.2	NC_044542.1
Lassi	1	TCTCTTACAGATGGGGGGCTAAGAGCTCCTCTGAGTGACCGGGAGTCTGGTATGCTGTGTCTGGGCTGCAGA				
Lassi	WeaningWt	TTGTCAAACAAAGA	gene	protein_coding	GCF_000803125.2	NC_044530.1
Lassi	WeaningWt	TTGTCAAACAAAGA	gene	pseudogene	GCF_000803125.2	NC_044530.1
Lassi	WeaningWt	ATGGGGTGAACCGT	gene	protein_coding	GCF_000803125.2	NC_044530.1
Lassi	WeaningWt	CAGGAAGCCCTGCA	gene	protein_coding	GCF_000803125.2	NC_044529.1
Lassi	2	TCGCCCGCTCAGAG	gene	protein_coding	GCF_000803125.2	NC_044531.1
Lassi	2	AGCCCCACGAGGGA	gene	protein_coding	GCF_000803125.2	NC_044545.1
Lassi	2	GTTTCCGGTCTGCTG	gene	protein_coding	GCF_000803125.2	NC_044526.1
Lassi	2	AGAAGGCCTCTGCC	gene	protein_coding	GCF_000803125.2	NC_044544.1
Lassi	2	GGGGACACGCGGCC	gene	protein_coding	GCF_000803125.2	NC_044544.1
Lassi	2	GTGCCCTTGCGGAG	gene	protein_coding	GCF_000803125.2	NC_044544.1
Lassi	2	tttttgaagaataacTA	gene	protein_coding	GCF_000803125.2	NC_044513.1
Lassi	2	GCAGCAGATCCATG	gene	pseudogene	GCF_000803125.2	NC_044526.1
Lassi	2	AAGtggggggagaga	gene	protein_coding	GCF_000803125.2	NC_044526.1
Lassi	2	ATGTaatttgaagaaggaaaatgcCTTTGCGTGGTCTATTAGTCAGatttctgcagagaaacagaatcagcagAA[C/T]GT				
Lassi	2	CCGCAGTCAGCAGTGAGGACCAGTCTGGCAGGAACACCCCGGTCAGCAGGCCCCACTCTGTCTCGGTGGGGA				
Lassi	2	CTCTCCAATCCTGTGGCGGCTGAGAGAAGTGAGGAAGCTCCAGAAGAGAAGATCGGAACGAGCAGAGGTT				
Lassi	WeaningWt	GGTGTGTCACGTGT	gene	protein_coding	GCF_000803125.2	NC_044513.1

start	end	symbol	GeneID
YGCCC[G/A]CAAAGTGAAACAGAGAGGCCAACGATGACAACGACTGCAGTCATTCACCATGGGACATAGCAAGAGAAAGCAATT			
29862630	30055565	ZNF469	105104694
12956529	13321456	GALNT17	105089692
32696972	32746339	SZT2	105090934
/C]GTAGAGAGGCGCCCTTGACGGCAATGACCGCCGTTCGGCTGCAGCAGACTGGGCCCACGTGACCAGTCACGATTC			
118857502	118924774	EVC	105085112
34848093	34857174	POMGNT1	105092917
92564763	92598595	BEND4	105089319
17834462	17865146	CD9	105098229
CAA[A/G]CATGGTAAATACACTACCGGGGTAGAGAGGCCTCTGCAGCCATCCAGGGACCCCAGGTTAGAAGCCTCTTCCACA			
32696972	32746339	SZT2	105090934
ACAC[G/A]CCTTCAATTTTCTACATGGTTAATTGTTTATCAGGCAGGTTGACACCGCAGTGGCTCACTGGGTGGAAGGTCTCT			
AG[C/T]GGGAACAGGACACATGgataaggagaaatgaaaacttgggCYCATTTTCTCTCTGCAGGTTCCAGGATGATGGAG			
13252142	13551204	NEDD4L	105105073
1685279	1831427	LOC105088769	105088769
9051985	9453579	THRB	105095623
31332478	31444474	CPPED1	105089030
ACCG[A/C]GACGCCGGGCAAGCTCGTGTGACGGTGCCTTGCTTCCGCTTTCACAGGTAAAGGGCCAAGTTTTAGGTGGAGCTT			
GTGGCTGCTGAGCACGTGGAGTGTGGCCAGTGCAGCTGAGGAAATGGGCAGTGAACTGCAgttaattgaaataaa			
/A]AGAGAGACGCCATGAGCCGTGGGAACCGGGCAGTGAGGTGCTGGTTCAGGACTGGCCCCTGTGGCGACAGGTTAT			
AT[C/G]AAGCTGCCAATTCCGTGAGAGCCCCTCATCACGCAGTTCCACGGTCAGATGGGTTCTGAGTCGTCGTTGACGCCG			
71659532	71932263	LOC105095098	105095098
33989548	33993270	SDR39U1	105102436
A/C]RGCCTCGGGCTCCCCTCTGCATTCACACCTACGAAGCCGGACGCTCTGCCCGACGACAGCCTCAAACACCTGAGg			
18044459	18258611	DTNB	105100036
18220337	18256741	OLFML2B	105092691
30157591	30204161	ZC3H18	105104682
30157591	30204161	ZC3H18	105104682
12788500	13054847	SLCO3A1	105085453
GGTAAGAC[T/G]GSAAGCAGGAGGGGTGGTTAGAGGCTGCTGCAGTAAGCCTGGTCAGAAACAACAGCAGCCTGAACTCCCCGATGA			
22007140	22402208	ADARB2	105100512
/T]GYCTGTGTTCAGAAAGAGGTTTGCAAGGAATTGGTGGTCTGAATAAACAGGCCCCCAAAGATGTTCATGTCCTCA			
A]tgatGTTGTGGTTGGAGAACTATTTCACGTACTAACCGTATTTCTCTGTGCAGGCCTGGTCTGCAGGGTTGGCAA			
92265343	92333620	SETD3	105095440
TTC[G/A]GAAGAGCTTGGATCAACTCACAGCGGGATGTTTTTCCCCTGGCCGTGGACAGCTTCCCTCCGCTGCAGGCCCTCC			
GTGC[G/T]GGGGGCAAAGGAGGCAMTGGCTTGTCCTGRGCAGGGGTGCAGCTCAGCAGGGGAAGCRGGACGTGTGTGAGCGTG			
GAAGC[G/A]GGACGTGTGTGAGCGTGGGGGAGCGGGGGGAGAGGAGGATAAACCCACCCGGGAATGTAAGACACGAGCAGCCTC			
1751030	2542845	NTM	105094287
1751030	2542845	NTM	105094287
4512674	4520522	PHF13	105100254
/G]AGAGCTGAGCACGTGACACCAGAGCCGGCTGGCCTCCGGTGCTCCCCTAACTCGAAGGGCTAGGACCCCTGTGAG			
1751030	2542845	NTM	105094287
CAA[C/G]CCTTCTTCCCAGGAGAGGCCACCTCCCCAGGCAGGTCCCAGCAGCCAgactYcttcctttctccccactgcATGG			
ct[C/T]cttcctttctccccactgcATGGTCCTCTCCGGCTCTTGGCAGCTTCCCAGCTGCTCAAGCTGAGCACGGTCCTC			
73640879	73655465	LAIR1	105087426
8926165	8986418	HOXC4	105094550
8950701	8964476	HOXC6	105094548
GT[C/A]GGGCGGYTGGAGGACAGGGTCCTGCGGGGACTCAGAACCAGCCCCCAGGTAGCAGTGCTTTGAGACCATGTGTGG			
TT[G/T]GGTTGCTTATTGGTCACCTTGACAGGAGCCATCTCTGCAGAGTGGAGAAAAGGAGGCCTGAATGGACTGGGTTAA			
TCC[G/A]TctgcctccaggcccagggcccgCGTGTTGATATTTACACCTGGACTGCCTTCCCCTTCCACAGAGCCTGCAGCC			
TGTCACCAGGTGGCTTCTCTCCCTTTAAGTATTGCTTTCTCGAATGCCACCTGGACATCCGGCCAGGACACTGG			
TCT[C/T]CTGCCATGTGATGGCTCTGTCACTTGCYTTGGCTCTGATTGGGCAAAGAGGGAAATAGACGTGTTGGTGCCGGGA			
GC[C/T]TTGGCTCTGATTGGGCAAAGAGGGAAATAGACGTGTTGGTGCCGGGATTTGATGTCCACGAACCAGAACAGGAAG			
GGG[A/C]CCGCCTCCGCGCCCGCCTCCGCGCCGCGTGGCCGTTGCTGCAGCAAACTGGCTGGGCGTCCCCCGTCCATCTGCT			
TGC[A/C]CTCCTCAGGCCCCAGCGGCCCTTCGGGMCCGCCTCCGCGCCCGCCTCCGCGCCGCGTGGCCGTTGCTGCAGCAAA			
25578932	25592596	PGAP3	105099040
AGg[G/A]ggctcctgcctcccagcttgcCTGGGAGTTGGCCTGGAGCGGGTGGGGCACCCATTCCAGAAAGGTAGACCAGAA			
44896799	44987128	SSUH2	105093179
30294321	30432726	LOC105089063	105089063
30294321	30432726	LOC105089063	105089063
30294321	30432726	LOC105089063	105089063
24237118	24237191	TRNAT-UGU	116148038
1939811	1943215	LOC116149858	116149858
1939811	1943215	LOC116149858	116149858
1939811	1943215	LOC116149858	116149858
G]ATGCACTGCTTTCTGGATGAAGGCTGAGAAAATCACAAGGTCTCAGGAGCCTCGCCATCCAAacaaggagaggtg			
11062841	11192940	ANKH	105090628

/C]CTGGAGGCCATGTGAAGGGGAGATCTGGGAGGACttccaggaggagctgggagagggatgTGGCTACAGGTAGGG

114471083	114598984	LOC116155034	116155034
3541869	3629314	LOC105091528	105091528

CAA[A/G]CATGGTAAATACACTACCGGGGTAGAGAGGCCTCTGCAGCCATCCAGGGACCCCAGGTTAGAAGCCTCTTCCACA

TAATT[T/C]TAGAAGGTGAGTTATCAGGAGCCACAGAGAAATGAATTGATTCACTTCAACTCACTGTGCATTGTGTTCAGTCCC

GCCC[T/C]GCACGCCAGCGCGGTTCCCGCTCGGCCGTGGCTCTGCACCGCGCACCGAACGGCTCTGGCTGCGACGACTGCTCG

AGC[G/A]GTGCGGGGGCTGgtccagcctgcagcccaggtCCCTGCAAGGAGGCTGGGATGCAGATGCACACATTCTCCACGG

TTCA[G/A]TTCTCAGACCACACAGGCCTGGGCTGTGCCTTCCCAGGAGGGAAGCRGTGCGGGGGCTGgtccagcctgcagccc

ACG[C/T]GCAGGGCCTGGCCCATCATGACTGATCTGCAGGTGAGGAAGGGCCTGTGAATGGCTGGGCAACTGGGAAGGCAGA

]TGTGGGCCTGGAGGGGGAAAAGGTTCAGGAGAAGGCTTGAAGGGGATTTTTGCACTGAARGTCACGATGTCCTGC

GT[A/C]GTCCACCCCAACACCTAGAAAGCAATCTTGACCTTTGTCTTAAATGCTCTGACTCGGGCAGCCGTGTGTACAGCA

7772932	7927015	COL26A1	105092396

GG[C/T]GCCCAGACCCAGCAAGGCTTGATTTTAGCATCGTGTGAACTAACGTGGCGTGAACTGTTCCATGAAAGACCCTCA

GAT[G/A]TGGCCACACTGCTCCACCCTGCCCCCCCTAGAATTCAGGYGCCCAGACCCAGCAAGGCTTGATTTTAGCATCGTG

79043468	79069369	CCR6	105101448

CG[C/T]GTGTGCTCACATGTGGGTGGGCTGTGTGCACGTGTCACTGTTGGTGTGTGCATCTGCATGCacgtgtctgtgcat

GC[G/A]GCCCACCCCCGCCTCCACGCGGCCTGCAGGCCCAGACCCAGCCCTGCTCAGGACCCACCTCTGCCAAAGCACAGA

CGTGA[G/C]GGTGTGCTGGTGGGAACGTGATCCATAAGCACAGGGTAGGCACCCACTGTTTACTGGGTATTTCCCTCCGGTGCC

758015	768786	LOC105099090	105099090

G/A]TTTATTTCCGTCGGAGCTGCGCCTGCGGCCCTCGGCAATCATCTGGAGCAGCTTTCCTCATCAGACTTTCTGCAG

GG[T/C]GGCTCTGAGGCTGTGTCTTCAGAATGCAAACAGGGTCTTTGTGAAGCTACCTGCAGCCCCAtccccgcccggccc

C[T/C]CATTTTCTCTCTGCAGGTTCCAGGATGATGGAGAGTCGTCCTTGATACCCATGgaggggttggttccaggacccc

C]cccMMMaWCCMCYCTTTTYMCCYCYCCYCTTGGGCACTCATAGTCCGGCTCCAACAAACCACCTCCTCTTGCACA

22579157	22667382	BCR	105095188
6001954	6026489	LOC116156028	116156028
3020069	3039900	SLC15A4	105094350
8724343	8926344	SRPK2	105093022
8083850	8437870	CUX1	105092384

CAGAG[C/A]GTGACAGGGCGGTGCCGGCGTTTCTGTAGGTGGTTTCAGTGAGTCGTGCTCAGAGCCCAGGGGGTGGCCAGCGGA

AGMataga[T/G]ggRgggRagggRRggggctctaAGGATGGGGCAtctgcagaggggctggggtggcagcTGAGCCATGAGAGATGA

1325183	1449304	COLGALT2	105096219
454275	547863	LRRC8D	105085533
50227062	50504416	BANK1	105089555

]CAGGGCTGAGTGATTCTTCCCTTTTAGGATTGTTCTTCCGTTTGTTTCTTGGTCTTTCTTGGCTGGACCGCCAGG

AGGGGT[G/A]CTGTCTTACACAGCTACCCCAGGTGCAGGGAGGGTGACTCAAGAGACGTGGACAGGAGCTTTGTGCCCTGCAGGG

81656209	81688229	LOC116155984	116155984
6177926	6317076	TMEM131	105099800

GCYG[C/T]GTCGGCTGAGCCTGCAGCTGCACGGGGATAAATATTTGCCTACAGCCCCATCTCTCCTAACGAGTGTTTGAGTGT

1751030	2542845	NTM	105094287
9075189	9228378	LOC105094554	105094554
65063094	65085295	TRMU	105091837

TGCT[G/A]GGAGAAGGTCAGGCCTCTGGAAGAACCCAGGGGAGGTTGGAGATCCAGTGGGagctgcagaggcagggctgcGGG

GGC[C/T]GTGGGCTGAGCCATGCGGAGCGGGGAGCGGGGCTGCCCGGCCTGGAGGGGAAGGTGTGGAAATCCGACACAAACG

58712349	58767563	TRIOBP	105091966

G[T/C]GGTGGCCCCTTCTCTGGGGCTCCTCACAGCCAAGCCCCTGCAGGTCTACCCTGCAGCTTCAGTCGCATAGCCGAT

TAAC[A/G]TAGGAACAGMGATGCTATAGGCATGTCTCAGAGCGCAGGGCAGCATCCAGGGAAGTTCTGACAGGAGGTGACCAG

ACAG[A/C]GATGCTATAGGCATGTCTCAGAGCGCAGGGCAGCATCCAGGGAAGTTCTGACAGGAGGTGACCAGGTGGGGCACT

61255789	61282289	ZER1	105092563
19940303	19949709	LOC116157202	116157202

acactctaccgctgagctctgcCTTCCCCCTTGCATTGCCTCTTTTAGCCCTCTACTCCCTGGCT

[C/T]GGAGCATCACGTTGTGTGCGTCACTGTGCCCACATCCCATTGTCCAGACTCAGCCACGTGCTCCTCCCTGCGTGC

TCCAGTGGCAGGAACTCAAAGTCCTAACTCTGCAGTTAACAGGCAGGACTCGAGGTGAAGACACAAGACACACC

1257019	1704075	OPCML	105094288
1351602	1575238	GABRB3	105090789

AGA[G/A]TTAAAAGAGGGTTTTGCCGCTGAGCGCACAGCCCCTGCAGAGCAGCTCGGGCGGGCGGCCTCGAGCGGCTCTGC

83010486	83031221	LOC116155679	116155679
12451670	12653709	TRERF1	105088645

C/T]GGCTCCACGGTGCTGTTCCGTGCGCCAGGCTGTGCTGCAGACGCAAACCAGTTTCAgcatttcctgtttttcttc

[C/T]acccccctctctctgcccatctctcTTCCAACTTGTCCCTGCCAAACCAAGGCAAARGGAAGCGCCGCACCTGTAG

4052551	4136871	KCNAB2	105104717

ACAGGATCTGCAGGAATAGAAATCAATCCTTCTCCTCAAGAGCCTGGACCACTGGCTCACTctatggagaaaatg

29034214	29053093	NGFR	105104837

ACT[G/A]GGCGATTCATCCGAACAGAGGACTATTGTCGAATGTYGAATGTCATTGATCGTGTAAAAATTACTGCAGTATTTA

T[G/T]CTTTCTTGGATTAGCAAGAGTATGAATGCGAAGGGCCTCAGGCATTTCTGAAACACCTTGCATCACTTTGGCCGG

62085760	62620370	FGF14	105094098

GCR[C/T]GGGCTGCTAATGGCTCTAGGCTGTCCTGGTCTCTGAAGGATTGCCCTGGGTTTCACTGGAAGGCTGTCCCCCACA

8743278	8779526	TNFRSF1B	105107099
1882265	2067317	GNG7	116148370

[C/T]GGAGCATCACGTTGTGTGCGTCACTGTGCCCACATCCCATTGTCCAGACTCAGCCACGTGCTCCTCCCTGCGTGC

17966567	19141623	TENM2	105089397
62907745	62966819	MPPED1	105091862
66595802	66738952	COL5A1	105106307
66595802	66738952	COL5A1	105106307
66595802	66738952	COL5A1	105106307
66595802	66738952	COL5A1	105106307
63187411	63215171	SULT4A1	105091860
26215302	26393949	RIPOR2	105089137

GTTAGAC[G/T]TGAGGTTGCCAAGGacaatgaatgaaagaaagcttTTCCAGAGACTCTACAAGTAAGCTGGAGTCCCTGTGTCTG

C[T/C]ACATCAACCGTCCTGGCCCACGGCCAGCATCACATCTCACGGCCTCTCCAGGAGACACTGCAGCCAGAAGGGCCG

12877531	12895509	ARMC6	105103710
12976787	12987958	TMEM161A	105103711

GGC[C/T]GTGGGCTGAGCCATGCGGAGCGGGGAGCGGGGCTGCCCGGCCTGGAGGGGAAGGTGTGGAAATCCGACACAAACG

58712349	58767563	TRIOBP	105091966

TAAC[A/G]TAGGAACAGMGATGCTATAGGCATGTCTCAGAGCGCAGGGCAGCATCCAGGGAAGTTCTGACAGGAGGTGACCAG

ACAG[A/C]GATGCTATAGGCATGTCTCAGAGCGCAGGGCAGCATCCAGGGAAGTTCTGACAGGAGGTGACCAGGTGGGGCACT

27420269	27422452	HOXB2	105095789

GGG[A/C]TCCTGAGTGCCTCTGGGAAAGGGTGAGGTCATCACTGAGAACCAAGAGACATTGCCACCCAGCTGGGGCTCACCC

17674829	17758337	BSN	105088160
4732249	4822922	MAP2K4	105095916
7183359	7458124	FRY	105101043
23326751	23663622	LOC105095261	105095261
35887024	36075217	NEDD9	105102362

ACA[T/C]GTGTGTGACCCTGCACATGGGCAGCGTGTACACTGCCTCTGCCAAGTGCGACCCCTGCAGGTGAGCACCCTCACC

at[G/A]ggaaatttggtGGCATTTTTACTTGCTCCTGTTACATCACCTCCCAGCCCGGTGAGACATGGTCTGGGGAAACTG

2210640	2425095	ANK1	105086428
2557157	2589983	DDC	105105292

C[G/A]CCTAGACGTCAGATCACTCAGTGCAGACTCCTCTCACTTAGCTCCGAGGCCCCCAGTCACTCCACCCAGCGCAGC

GAC[A/G]TGGAGTTTAAGTCGGCGCAGGCGGAGGGCGTGCAGCCGCGGTGGGGGACTGAGTGACCGACAGGAGCCGAGGGC

1858125	1860191	GADD45B	105103609
22019334	22741078	GRID1	105092491
1325183	1449304	COLGALT2	105096219
5703078	5802406	PAQR8	105099529
5736472	5749959	LOC105099528	105099528
77814736	79826534	CNTNAP2	105095041

GAC[A/G]CAAGCRGAGGACACGGGGGTTGAAGCTGCAGCAACCAGAGGCCAAGTTTCAGGGGATTTTGAAGAATTCCTCACT

1339146	1428504	NEK1	105101615
30438029	30460725	LITAF	105089040
51901233	51948361	ALDH1L1	105104512

G]TCACTTGCCCCGTCCACGCTGCTCTGTCCCCGCCCCGGAGGGCTTCCCACTGCGAGGCGGGCTGCTGCTGTGGGC

/A]CAGCTCCAGTGTTCATGCCCTAAATTCAGATCCGCTACAAGCCKCTGGCTGTGCTTCTAAACACCCCCAGACTCT

1325183	1449304	COLGALT2	105096219

CGC[C/T]CTCGTCCCTGTTCTTGACACCAAGCGAAGCTCCCGTCTTTGTTCCGGCTGTCTGTCCTGATGCTCTTGGAGGCGC

4225299	4289837	LNX2	105086578

AG[T/G]GTAAAGAGAAGAGCCTCACACTGAGAAAAGCCCTCGAAAAGCGACCTGGCGCGCCCCGGGCTCAGAGCCGGGAGC

27775247	28189597	GSE1	105098913

TGT[C/T]GAATGTCATTGATCGTGTAAAAATTACTGCAGTATTTACAGTGTATTAATTGGTTTGTAGACCATAAATACATGG

CAC[G/A]ACTTCACTRGGCGATTCATCCGAACAGAGGACTATTGTCGAATGTYGAATGTCATTGATCGTGTAAAAATTACTG

CAA[T/C]GGAGATTACACRACTTCACTRGGCGATTCATCCGAACAGAGGACTATTGTCGAATGTYGAATGTCATTGATCGTG

84340528	84498084	INPP5A	105104581

GAA[T/C]GTGTGTTAAACCTGTAAGTAGCATCTTTCACCTCACACGGCTCCCGGtattcaattaaaacaaaaaccacctgAC

73417150	73515729	LOC116154664	116154664
17016638	17083334	SPIN1	105086413

TC[T/C]TCAAAGCTCAGACACCCAGCGCCCTCACAGGCCCCGGTGCTTACAAGTTTAATCTTCATGAAGGATTTAATTGGA

GC[A/G]CTGTCACTGYGCCTCGTCCCATCTCCTGCAGGTGGGCGCAGTCTcagggcctggctctgggctccaCCGCCTGCC

68286451	68298406	LOC116156723	116156723
66595802	66738952	COL5A1	105106307

CAGC[G/A]ACTGCAAAGATGAGCTTTCTAAGCTGACTTACCTKAGTGATTCTTCCGTCTCCTCaatgtacagatgaggaaact

AGWCA[C/T]GGCTGTCATCGGGCAGGAGCGAAGATTCACCAAGGCCCTTCCCTGTGCCAGCTCTGtgccggggctgggggccca

74027041	74033016	DNAAF3	105098737
5565835	5638407	TRAM2	105099545

]cctctKaaRCCARAACCAGgagctgcctctcctctctccctgctctcgcctcctctgcctccaccccagcACC

4259447	4349980	ACOT7	105104722
4259447	4349980	ACOT7	105104722

CTAA[G/A]TGCCCCTCATCCCACCCTCACTTCCAGCCCCCCGGGGGCTTTGCTCTTGGTCCCATGAATGTGCTGTTCCAGcaa

TGGG[G/A]AAGTCGTGCAGTCATCACACATTTCTTAAGCGCTAGCTCTGGGCTGCACTCTAGCTAAGTCGTGGAGACGCAAAG

CTT[A/G]STTGTTTTTGGATCTTTGATGAATCACTTTAGTTccctgagtctctgtttccttgtctgcagAAYAGTGTGATAA

TACAA[G/A]GGAAACACGTCATTCTGAGGCGCAGAACAGGTGGAACGTAACGCGAGCTGCAGGAAAGACAGGCgacctggagct

24420245	24441500	LOC116149382	116149382

C/G]TGGCCACTAGctgcaggggaaggggctgtggggcAGCTCCAAGCTGGACGCCAGAGCCCTCTGCTGCGTCCTGTT

60802496	60833647	SLC25A25	105092588

CAC[C/A]GTGGGGAGACACGCTGTGTGCAGGACCTTACTAGGGGACAGTGCCCTGGGCCCCcggggagagggaaggacagaC

GAG[C/A]CTTACCAGCTCCAGCTCTAGCGCTAAGTCATGAGCCGGAGCGCACACCAGCGTCAACTTCTGCCCCAGCTCGGAA

13466587	13546554	LYPD1	105089072

AAGG[T/G]GGCCATTCCKGGSAGRSGGGRGGGGCSGCATCTGRSGKGGAKGGGGGGGTCTSGKGGAGGGKGGGGGACGTGCTG

TTT[C/T]CCGGATCTCACACACCTTGggagaaatatataaaaaagcaaTTCCACTTCTTTGGAAGAGGAGAGAATAGGGAGA

C[A/T]CACACACTTGCSCTTGCCAATCTAGCACCACAGTTTATTCACAGGCAAGTAAATCAGGACTTCTGCTTTYCCGGA

501050	677444	HERC2	105090766
7326926	7518906	CLASP1	105088283
551849	653674	ADAP1	105102058

C[T/C]CGACACGCACGGGGCCAAGCACGCGTCAGGCCCCGCGGGACGTGGTGCGGACGGGACAGCAGATCAGACAGGCCG

32859602	32996832	PLEKHA6	105098648
32859602	32996832	PLEKHA6	105098648
1007560	1010748	LOC116148887	116148887

GGTCTGAGGCTTTGGACACTGCAGACGCCTGGGCCACGCCTGCAGCAGTGAGGGTACAAAATCCGGCACCTGG

1751030	2542845	NTM	105094287
6737897	7020790	LOC105106140	105106140

AACAT[T/C]GTACTTGTCCTTGCAGAGTAATGCAGAGCTGACCATCATTCACAGCATCAGAAGTTGAGTCATACAATGAGCTAT

73170788	73283143	ESRRB	105086076

AGG[G/A]TAAGGATGTCCCTCACCTCCAGCTGGACAAAGGGACGTCACCACGACCMCTTGGAGAGCTTGCTCTGAGTTCCCT

TCCC[A/G]AGGRTAAGGATGTCCCTCACCTCCAGCTGGACAAAGGGACGTCACCACGACCMCTTGGAGAGCTTGCTCTGAGTT

3042023	3219688	RFTN1	105104367

ATT[A/G]TTTTGTTCAGTATGTTCGGGCTGTTCTAACAAAGTGGCACAGGGTAaagtggcttgtaaacaacaggaATCTGTT

CT[C/T]TCTTGAYCTCACCCGAAGCTGTCTGCAGAAGGTTTCCAGACAGTGTCTCATCCTTTCTTCACAGGGTCATTAAGT

AG[T/C]TGCTGTTCAGCAAGCAGGTGGACTGATCTCGGGTCAAGTGCCCATCTCCGGCCGTTATGGGGAGGTCACGTGGTG

57112336	57120921	GMNC	105097551
59898527	59952690	SMCO4	105100553
59898527	59952690	SMCO4	105100553
59898527	59952690	SMCO4	105100553

T/C]gtaaaagtgaaaatctgCATGAATGGCATTTGTTTTCCTAAGCAAACACCCGATTAGGTCCTTTTCCCTGGCTTC

AT[A/C]AATCTGTGGTCAAAGGGGGgtcaagatggagaaaaatatctTCTGCAGATTCATTTAGAAAGTAACTTTTTGAAT

TG[C/T]TGGCAGGGGCTGAGTCCCCTCCCCTGCACAATMAATCTGTGGTCAAAGGGGGgtcaagatggagaaaaatatctT

TGTG[C/T]AGCTGCTGCAGCCCACGCACCTGGGCTGGGTGGCAGGAGGAGGTGGACCCCATCCTGATGCTAGCTGAGCAGGGC

GTGTCAACCCAGGCTGTGCTTGCACCCCWCTCTCACCGCAGCCAACATACACTGCCTGCTTCCTCCGGCCCAGAG

CCC[T/A]CTCTCACCGCAGCCAACATACACTGCCTGCTTCCTCCGGCCCAGAGCATGCCAGGCTCACTGCCTTCAGGGTGGA

121836315	121959561	HTT	105103925

CTCTTTGCCAGCTCCATGCTAGGTGCCCAGAACAGGACCCCTCCCCATGCAGCCTGGCTCCCGGGGCACCCGAG

1762624	2231818	CDH4	105095666

AAGC[A/G]GAGGACACGGGGGTTGAAGCTGCAGCAACCAGAGGCCAAGTTTCAGGGGATTTTGAAGAATTCCTCACTTGGGAG

3192899	3354050	PHACTR3	105095663

ARAGA[C/T]GTCTTCTAGGGCAGTGATGTTGAGCCCAGCTCtacaagtggaRaagaaaaggggggMaagaacatTTTAATCCAAG

ACCCG[C/T]GCTGACAGTGACAGTGTGGTGGTGTGGGGGACGTAGGCAGTGCAGGCTGTGAGGCTTGACGTCAGCACCCCCATT

22055922	22064231	ATF6B	105090854
27821402	27826080	LOC116148488	116148488

]cctgCYCYAGATTTCCTTAACAGCATCACTAGAATGCCCGGGACCAACAGTGGAAAGAAACCACCTACACTATGT

CTGG[A/G]AAGGGGGGATTAAATTGTGGCATGAGCAGGTAGGAGATTAGAGAATGTTTTACCGTGAGCCTGGCACGTTCTCCG

32859602	32996832	PLEKHA6	105098648
32859602	32996832	PLEKHA6	105098648

MAAGRG[C/A]AGGTGAGTCKGGAGAGGGATGTAGMatagaKggRgggRagggRRggggctctaAGGATGGGGCAtctgcagaggg

ACKGC[C/A]AGAAGKGACMAAGRGMAGGTGAGTCKGGAGAGGGATGTAGMatagaKggRgggRagggRRggggctctaAGGATG

2811826	2839378	LOC105085751	105085751

YGTTGGGTGACATGTCTTTGGGCTCCTGCTGCCGCCTGTGCCGTCCCCATCCCAGCGAGAGCACAGGG

GTGACATGTCTTTGGGCTCCTGCTGCCGCCTGTGCCGTCCCCATCCCAGCGAGAGCACAGGGATGCGAGA

CA[G/A]GGTTCCAACTGCAAGTGTCCTGCTGAACAAGGCAGAAGCTGCATGGTCTTCAATGACCCAACCTTGGAAGCCTCA

/A]GGGAGAGTGTTCCCTGCAGTGCTcaYggggcagggagaagaggatTTGTGAGGGGTGGGAAGTAGAATGAGTCCT

29453526	29478318	LOC116148938	116148938

G[G/T]CACCTAATAGCGCTTTCtatcagttttcccatctgcagaatggggacTGCGCTTCTGGGCAGACAGGGTGGTGGT

GTG[T/C]GAGGGGCTGTACGTGGCTTGTCTCATTTAGCCCTGCAGACAAGCCTGTGGGGCAGCATACGGTCCTGTGCTRGTC

GCMCSCCCCMGCTGTATKCGCTCCTTCSCCCYCTTTTCCCCCAGTCACACCCCACTCTTTCCCGGCAGCTGT

27704955	27745802	ERICH1	105084399

[G/A]GGGGATAAGGTCATTTGAGGCCGCAAGCACCAGTGGTCCAACTGGGAGACCAGGTCTGCAGATGTACAACGAAGG

6416246	6489725	FAM178B	105093685
18851644	19016203	MAPK4	105103420
220736	242854	CHFR	105101630
6468064	6536940	PATE3	116150179

GCCC[G/A]CCGGGGGACCGAGCCtgcctctccctgagcctcaatcTTCTGTCTGATGAGGGTGATGCCttagagaaaaatgtt

MG[T/C]GCAGAGRCTGTCAGGGCCAGTTACTATCACAGATCTGCAGTTCCTCACCTACAGTTCTGAAAGCTCAGTCTGAGA

TC[C/A]GYGCAGAGRCTGTCAGGGCCAGTTACTATCACAGATCTGCAGTTCCTCACCTACAGTTCTGAAAGCTCAGTCTGA

]GGGGGCAGTGCCTGGGCAGACTCMGYGCAGAGRCTGTCAGGGCCAGTTACTATCACAGATCTGCAGTTCCTCACC			
22019334	22741078	GRID1	105092491
22019334	22741078	GRID1	105092491
22019334	22741078	GRID1	105092491
22019334	22741078	GRID1	105092491
AGT[G/T]GGAACTTGATCTCAGAACTGCAGTGCTGGCTGAGCAGCATATTCGCTTTTCTTAAATCTTATGGGGACTGCTTAC			
TG[G/A]GTCTGTCAGTCCTGTAGAGCCATCACCCATTGACAAGACAAACAACCCTGCCTCTGGGCCGGAGCATCAGCGGGG			
GTGA[G/A]AGCAACTTTGTATRTTAACCCAACTGGAGGAGAAACCATCCGGGACTGTGTGAAGAAGAGGCAGCAACACTGCCC			
A[G/A]TGTCTGCTCCAGAAATTCTGCAGCTAGAGTCAGTGTCTGTTGCTGGGGACCCCGACAAAGAAGCCATCAACCCCT			
2584367	2654959	CMC1	105087207
CCACTTACCTGTTCATAACAGAAGGCTGCAGAACGCAGTGCGTCATTTGCCATGGTCATGTTTTTT			
97315183	97331860	GAL3ST2	105096206
GATC[T/C]TAGAGCTCAAGGAAGTAGAAAACAGCTCGCTGCATTCCTGCAGAAGTGCCGGGTTTGCACATGTCAGACCAGCCA			
85747720	86129284	PTPRN2	105088983
85747720	86129284	PTPRN2	105088983
86425716	86475332	WDR60	105101917
14765475	14830230	SLC38A1	105097082
12633587	12868070	IGSF21	105103989
A[G/A]ACGTCAAGCCTCCAGAGCCGTCCTCGgacctttgttcctgcagcttcCACGTCCCTCTGTTTCCCCCACGCCTTT			
CC[A/G]TGGATCCTGAGATCTGGACAGTGCTGCAGGGATCCAGGTTGCACGGGAGCCTCCAGCAGCCTCTTGGATGGGTTA			
8743278	8779526	TNFRSF1B	105107099
C/T]TTGCTGCCCGTGACCTTGTTTCATGCTCACAACACGCCTAAGAGGGGTCTCATCAGCACCGCAttttcagaggag			
12902929	12951484	LZTS1	105102884
ccatcaCACCCAGCTGCTCCCTGCAGTGCAATGGTGAGGGGGCAACAAGAAATGCCATTCTAGCCAGGCTGCTT			
608783	655149	LOC116147128	116147128
73539964	73896869	HECW2	105088797
14127637	14175865	ROR2	105094547
AGCG[C/T]GGATGCTGCAGACACGCCGCCCCCGYGGGGCACTAACTCGCCAGTGCCGCCCCGAGGCAGTGAACTCATCTCCAG			
3457176	3494789	DPP9	105091778
31114918	31226318	LRIG1	105095248
3541869	3629314	LOC105091528	105091528
5703078	5802406	PAQR8	105099529
AGCC[G/A]AGCTGCAGGGCCCAAACCACATACCACAGTGGCGACGCATAGGAAGCCCCACGCGGCTGTGCAAATATGACTTCT			
33500309	33710664	RNF220	105090890
42114438	42379599	RALGAPA2	105101590
CCTCAGGGATGCAAGGGTGTCTTCAGGGGTGCGAGGGTGCCCTCCCACCATAAGTTCCGTGCCTGCCGGTCCTCC			
1850199	2031244	NMNAT2	105096225
68298906	69000741	EXOC4	105085399
20178164	20256322	NF2	105099460
ATTT[G/T]CAGAGCCACAGACAGGTGCCCAGACTTCCCACCCTTGTCATCCTTACCTGGAAGGGCCTCAGAGCCCCCTGGAGC			
5703078	5802406	PAQR8	105099529
5736472	5749959	LOC105099528	105099528
5802673	5819517	MCM3	105099527
42114438	42379599	RALGAPA2	105101590
29862630	30055565	ZNF469	105104694
11589160	11590071	LOC105090602	105090602
37428263	37446542	KIF18B	105094413
17611331	17615665	GAPDH	105098219
17611331	17615665	GAPDH	105098219
17644434	17645792	MRPL51	105098220
66394549	66465147	ELL2	105100891
38113766	38125628	LOC116157935	116157935
37832782	37843163	FAM171A2	105094445
ATGCGCACAGATACAGTTAGATTTGTTTTAAGGAAGTGGCTGATGTGATTGTGGGGGTCGGCAAGTCTGCAAT			
ACGC[G/A]CCRCAGTGTGAATTAGGTCATACAGACAACACCTCCAGCGACAGACAGTCRCCAGTAAGTCGCCTGCAGGATCTG			
TGGTTC[T/C]TGAGGTTTAAGAAAAGACGCCATCTCCAGAACATGGAAGTGCAGGCGGAGCAGCCAGKGCTGACGTGGAAGCTGC			
22822432	23201391	PDZD2	105087024
