# Supplementary material for: Genome wide association study for growth in Pakistani dromedary camels using genotyping-by-sequencing
Source: Anim Biosci. 2022 Nov 14;36(7):1010–21. doi: 10.5713/ab.22.0181 (PMC10330985; doi:10.5713/ab.22.0181)
Supplement: Supplementary Table S2. — The PDF file ‘Supporting Table 2’ contains a list of SNP associations mapped in the current study with SNPs that were within 1 Mb of those mapped in the study on Iranian breeds of dromedary by Bitaraf Sani et al [12]. [file ab-22-0181-Supplementary-Table-2.pdf]

Genome-wide association study for growth in Pakistani dromedary camels using genotype-by-sequencing

Sajida Sabahat, Asif Nadeem, Rudiger Brauning, Peter C. Thomson and Mehar S. Khatkar

Supporting Table 2. GWAS significant associations detected in the present study (Pakistani breeds) which are within 1 Mb of a significant association reported by Bitaraf Sani *et al.* (2021) (Iranian breeds).

Chromo	Pakistani breeds		Iranian breeds		Diff (Mb)[c]
	Position (bp)	SNP ID[a]	Position (bp)	SNP ID[b]	
7	83,006,915	22446_42	82,468,596	S7_82468596	-0.54 – -0.52
			82,483,675	S7_82483675*	
			82,483,685	S7_82483685*	
11	71,663,573	137595_71	70,990,227	S11_70990227	-0.67 – +0.69
			71,992,855	S11_71992855	
			72,060,113	S11_72060113	
			72,350,029	S11_72350029	
			72,356,390	S11_72356390	
			72,356,400	S11_72356400	
			72,356,401	S11_72356401*	
			72,356,436	S11_72356436	
11	81,674,156	103229_45	81,210,420	S11_81210420*	-0.46 – -0.71
	81,918,150	34447_68			
18	30,302,544	56307_46	29,830,420	S18_29830420	-0.61 – -0.20
	30,305,338	56308_47	29,958,631	S18_29958631*	
	30,307,339	56310_55	29,963,432	S18_29963432	
	30,438,158	56328_38	29,963,702	S18_29963702	
			29,985,605	S18_29985605	
			30,061,144	S18_30061144	
			30,075,711	S18_30075711	
			30,105,059	S18_30105059	
25	1,009,197	157643_51	262,766	S25_262766	-0.75 – -0.75
			262,898	S25_262898	
			263,079	S25_263079	
			263,086	S25_263086	
31	16,003,963	79310_43	16,977,384	S31_16977384	-0.08 – +0.99
	17,061,932	115825_14	16,998,649	S31_16998649	
33	4,172,256	83833_55	4,467,956	S33_4467956	+0.30

* Associations with $p < 0.001$ reported by Bitaraf Sani *et al.* (2021), otherwise $p < 0.05$.

[a] SNP ID from present study

[b] SNP ID from Bitaraf Sani *et al.* (2021) study

[c] Difference in SNP positions (Mb), calculated as (Iranian pos (bp) – Pakistani pos (bp)) /10^6.
